# Phenomenological Model for Predicting the Catabolic Potential of an Arbitrary Nutrient

**DOI:** 10.1371/journal.pcbi.1002762

**Published:** 2012-11-01

**Authors:** Samuel M. D. Seaver, Marta Sales-Pardo, Roger Guimerà, Luís A. Nunes Amaral

**Affiliations:** 1 Department of Chemical and Biological Engineering, Northwestern University, Evanston, Illinois, United States of America; 2 Interdepartmental Biological Sciences Graduate Program, Northwestern University, Evanston, Illinois, United States of America; 3 Departament d'Enginyeria Química, Universitat Rovira i Virgili, Tarragona, Catalonia, Spain; 4 Institució Catalana de Recerca i Estudis Avançats (ICREA), Barcelona, Catalonia, Spain; 5 Northwestern Institute on Complex Systems, Northwestern University, Evanston, Illinois, United States of America; 6 Howard Hughes Medical Institute, Northwestern University, Evanston, Illinois, United States of America; The Centre for Research and Technology, Hellas, Greece

## Abstract

The ability of microbial species to consume compounds found in the environment to generate commercially-valuable products has long been exploited by humanity. The untapped, staggering diversity of microbial organisms offers a wealth of potential resources for tackling medical, environmental, and energy challenges. Understanding microbial metabolism will be crucial to many of these potential applications. Thermodynamically-feasible metabolic reconstructions can be used, under some conditions, to predict the growth rate of certain microbes using constraint-based methods. While these reconstructions are powerful, they are still cumbersome to build and, because of the complexity of metabolic networks, it is hard for researchers to gain from these reconstructions an understanding of why a certain nutrient yields a given growth rate for a given microbe. Here, we present a simple model of biomass production that accurately reproduces the predictions of thermodynamically-feasible metabolic reconstructions. Our model makes use of only: i) a nutrient's structure and function, ii) the presence of a small number of enzymes in the organism, and iii) the carbon flow in pathways that catabolize nutrients. When applied to test organisms, our model allows us to predict whether a nutrient can be a carbon source with an accuracy of about 90% with respect to *in silico* experiments. In addition, our model provides excellent predictions of whether a medium will produce more or less growth than another (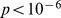) and good predictions of the actual value of the *in silico* biomass production.

## Author Summary

The ability of microbial species to consume compounds found in the environment to generate commercially-valuable products has long been exploited by humanity. The vast untapped diversity of microbial species offers a wealth of potential resources. However, little is known about most microbial species. While the metabolic network of an organism can be studied to find its nutritional requirements, we lack a reliable metabolic reconstruction for most species. We use *in silico* organisms to systematically explore whether an arbitrary nutrient can stimulate growth as a single source of carbon, and how effectively it can be used by the organism. We find that we can predict whether a nutrient is a source of carbon and the biomass yield of that nutrient with a simple model that transcends the diversity of species and their environments. Our model for catabolic potential can therefore be used as a baseline model for any microbe for which we lack a metabolic reconstruction.

## Introduction

Predicting microbial metabolism under a broad range of conditions would enable us to leverage microbes for applications in critical areas such as energy production [1], pollution amelioration [2], [3], bioengineering [4], physiology or medicine [5], [6] to name a few. While systematic *in vivo* growth experiments could in principle fill the gaps in our current knowledge, those experiments are time consuming and contingent on the ability to grow the microbial species of interest in the laboratory [7]. As a consequence, only a small number of microbes have been studied using these techniques. For example, Biolog (http://www.biolog.com) provides Phenotype Microarrays that have been used on species such as *Escherichia coli* [8] and *Bacillus subtilis* [9] but to date there are only 200 publications listed in Biolog.

To circumvent experimental limitations, a number of mathematical models have been developed aiming to predict microbial growth rates [10]–[14]. However, these models are only valid for a limited number of specific nutrients and are not easily generalizable because of the need to determine many parameters empirically. Indeed, developing such a theory seems an insurmountable challenge given the combinatorial number of possible growth media and the large number of unknown parameters such as reaction constants and enzyme affinities that control metabolic reactions [15]–[17]. For instance, the *in silico* reconstruction of *E. coli* contains more than 2,000 reactions [8]; taking into account that each reaction has at least two and up to tens of kinetic parameters [18], a detailed kinetic model would have on the order of 10,000 parameters, unless some approximations valid under certain conditions are made to reduce this number [19].

An alternative approach that is gaining in popularity is the development of thermodynamically-feasible metabolic reconstructions that can be used to predict the growth of individual organisms using constraint-based methods [8], [9], [20]–[27]. Researchers fine-tune these reconstructions to match the conditions of specific growth rate experiments, such as nutrient availability and ATP maintenance. These reconstructions, built by literature mining, can, under certain conditions, accurately predict the impact of individual nutrients on growth as sources of carbon, nitrogen, phosphorus and sulfur, thus allowing researchers to evaluate the demands of biomass production and to investigate how individual nutrients can meet that demand. While building metabolic reconstructions for new organisms is quite challenging, nowadays the process is becoming more and more automated [28]. Nonetheless, building a reconstruction still requires manual and/or experimental tuning, which hinders the generalization of these models. An additional caveat of these metabolic reconstructions is that the complexity of metabolic networks prevents researchers from obtaining an understanding of why a certain nutrient yields a given growth rate for a given microbe.

In order to formalize the intuition used to build and fine-tune the constraints used to predict growth rates using metabolic reconstructions, we present here a systems-level *phenomenological theory* of microbial metabolism, that is, a theory that yields a mathematical relationship between the maximal biomass production of a microbe and the set of available nutrients acting as carbon sources, without taking into account any microscopic details of the processes occurring inside the cell. The biomass production predictions of our model depend exclusively on the characteristics of the available carbon sources and the set of metabolic pathways that can catabolize them. Our model is able to match the predictions of flux balance analysis with no significant computational effort and providing insight into the determinants of catabolic efficiency.

### Background

Our phenomenological model expresses the impact that different carbon sources (or nutrients) have on a microbe's ability to grow using only information on the chemical structure of these carbon sources and on the ability of a microbe to catabolize these nutrients. Our model is built to reproduce the predictions of flux balance analysis calculations (FBA) on metabolic reconstructions, thus it will suffer from the same limitations. Indeed, while FBA is a powerful tool to investigate microbial metabolism and microbial growth in particular, it has a number of limitations when estimating growth rates and the effect of media and environmental conditions on growth.

It is well-known that microbes need a minimal medium and a carbon source in order to grow. Minimal media have been described for a number of species and contain essential chemical species without which the species would not be able to grow [20], [29]. The growth rate of a microbe, however, depends on many other factors including the uptake rate of nutrients, temperature, regulation, the availability of oxygen, etc.

FBA is a linear optimization method that predicts the maximal conversion of a set of carbon sources into biomass with a fixed minimal medium. In order for FBA conversion rates to reproduce empirical growth rates, one needs to consider an additional ATP maintenance flux which is obtained by fitting FBA results to empirical growth rates obtained for a certain temperature, minimal medium, and carbon source uptake. While ATP maintenance rates obtained for a specific minimal medium have been shown to give accurate predictions of growth rates in different minimal media for some organisms [8], in principle one cannot assume that they are valid for predicting growth rates for arbitrary minimal media.

Additionally, because metabolic reconstructions do not consider regulatory constraints, FBA will predict that a microbe is capable of uptaking two different sugars simultaneously, while it is well-known that if there is more than one sugar carbon source at high enough concentrations, the organism will exhaust the preferred one before consuming the others [30]. A microbe will, however, consume multiple sources of carbon other than sugars simultaneously and as a consequence grow faster [31]. It has been shown that steric constraints can already reproduce diauxic growth in some organisms in the presence of multiple sugars [32], however, there is no general framework that is able to deal with this issue when using FBA.

To develop a phenomenological model that is able to reproduce maximal biomass conversion rates per carbon source under aerobic conditions, we investigate the maximum amount of biomass that can be produced by an organism in the presence of a minimal medium, oxygen and one or more carbon sources including at most one sugar. To this end, we run FBA on metabolic reconstructions in which we remove ATP maintenance [27] (see Methods for specific details). We concentrate on a training set of four species for which there are high-quality metabolic reconstructions available, and which cover a wide range of microbial phylogeny: a gram-negative bacterium (*E. coli* [8]), a gram-positive bacterium (*B.subtilis* [9]), an eukaryote (*Saccharomyces cerevisiae* [21]), and an archaeon (*Methanosarcina barkeri* [24]). We validate our model on a test set of three species for which we also have high-quality metabolic reconstructions—*Helicobacter pylori*, a gram-negative bacterium [20], *Staphylococcus aureus*, a gram-positive bacterium [22], and *Mycobacterium tuberculosis*, an acid-fast gram-positive bacterium [23].

The rationale for choosing a small number of species for model building and validation is that lower quality reconstructions are likely to have significant gaps that could incorrectly bias the determination of the model. Additionally, the aggregate set of nutrients available for these reconstructions is of 352 nutrients, which cover all nutrient types and 90 out of 97 pathways available in KEGG [33]–[35], thus ensuring that our model is comprehensive.

We believe that the development of a systems-level model of microbial metabolism is not only complementary to current approaches but offers some advantages with respect to them. Specifically, while FBA run on metabolic reconstructions already has the capability of predicting maximum biomass yield, our model has the advantage that since it does not consider microscopic details of the metabolism of a species it is directly applicable to any other organism growing under the conditions of our analysis. In fact, we show that solving over a thousand linear equations under constraints can be well approximated by a simpler model whose principles are easier to understand. As such, our model offers the possibility of uncovering universal features of the metabolism of organisms that other computational approaches are not capable of. The mathematical model we develop is thus a valuable tool from both fundamental and applied perspectives, since it can help understand the metabolism of organisms for which a metabolic reconstruction is not available, or guide the process of validating metabolic reconstructions.

## Results

**Figure 1:**
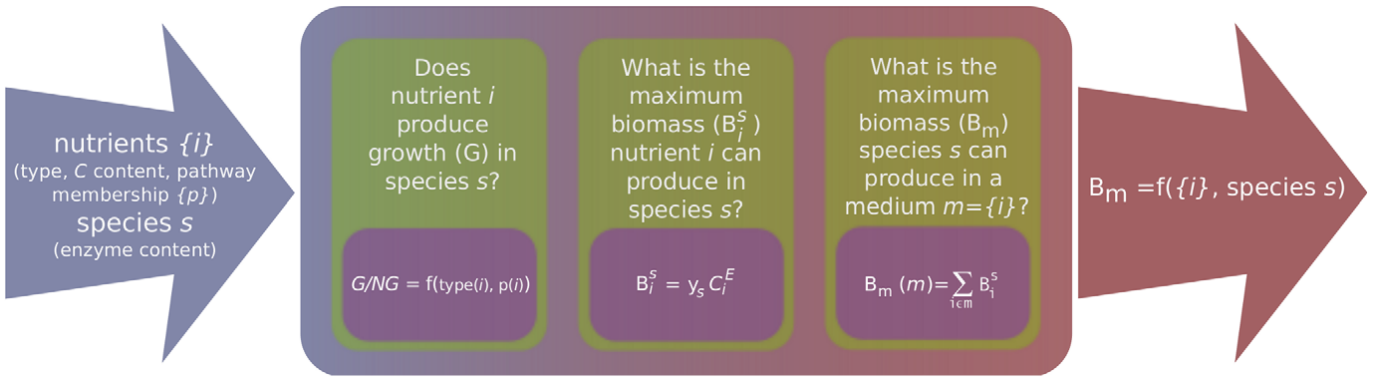
Schematic representation of the development of a model for maximum biomass production in complex media of microbial organisms. We aim at developing a phenomenological model to predict the maximum biomass production *B*_m_ of species *s* when growing in a medium containing a set of nutrients {*i*} acting as a carbon source under aerobic conditions. That is, we want to express *B*_m_ as a function *f* ({*i*}, *s*) that only takes into account data related to: i) the set of nutrients {*i*} available, namely, nutrient type, the set of pathways {*p*(*i*)} that can catabolize each nutrient, and the carbon content C*_i_* of each nutrient; and ii) the species *s*, specifically the presence or not of certain enzymes in a species that allow to catabolize specific types of nutrients (enzymes EC: 1.1.1.35, EC: 2.3.1.16, and EC 3.5.2.17 - see text). In order to achieve our goal, there are three different questions we need to answer: i) Does nutrient *i* produce growth or not in species *s* when acting as the sole source of carbon? We find that whether nutrient *i* produces growth (G) or not (NG) is a function of the nutrient type (see text) and its pathway membership; (ii) If a nutrient produces growth, what is the maximal biomass 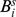 it can produce in species *s* when acting as the sole source of carbon? We find that 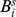 is proportional to *C_i_*, the number of carbons in nutrient *i*, and that the proportionality constant *y*_s_ depends on the species *s*. iii) What is the maximal biomass production *B*_m_(*m*) when growing on a complex medium *m*? We find that *B*_m_(*m*) can be well approximated by adding up the individual contributions 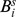 of nutrients *i* present in medium *m*.

In order to develop a mathematical model that relates nutrient (carbon source) uptake in complex media to biomass production, we need to address three different questions (see Fig. 1). The first question we need to answer is whether a nutrient can be a source of carbon or not. This is to say, first we need to develop a mathematical model that determines whether a nutrient can produce growth or not based on the information available for that nutrient and organism. Note that because we are interested in a binary output (growth/no growth), our model will depend exclusively on the composition of the nutrient and the pathways that can catabolize this nutrient.

Then, we need to assess what is the maximum amount of biomass a nutrient can produce when acting as the sole source of carbon. Our specific aim will be to find a mathematical relationship between nutrient composition (in our case, carbon content) and the biomass produced per unit of nutrient uptaken.

In third place, we need to assess how maximal biomass production is affected when several nutrients are present in a medium. That is to say, we need to determine the relationship between biomass produced and the characteristics of the nutrients available in the medium.

 Finally, to prompt and aid experimental studies, we use our model to predict which nutrients can be a source of carbon for four species lacking a metabolic reconstruction, and predict the biomass production of these species in complex media. We show that the carbons available in the 20 natural amino acids in a medium provide the best boost to biomass production regardless of the species.

### Catabolic potential: Can a given nutrient be a source of carbon for a specific microbe?

**Figure 2:**
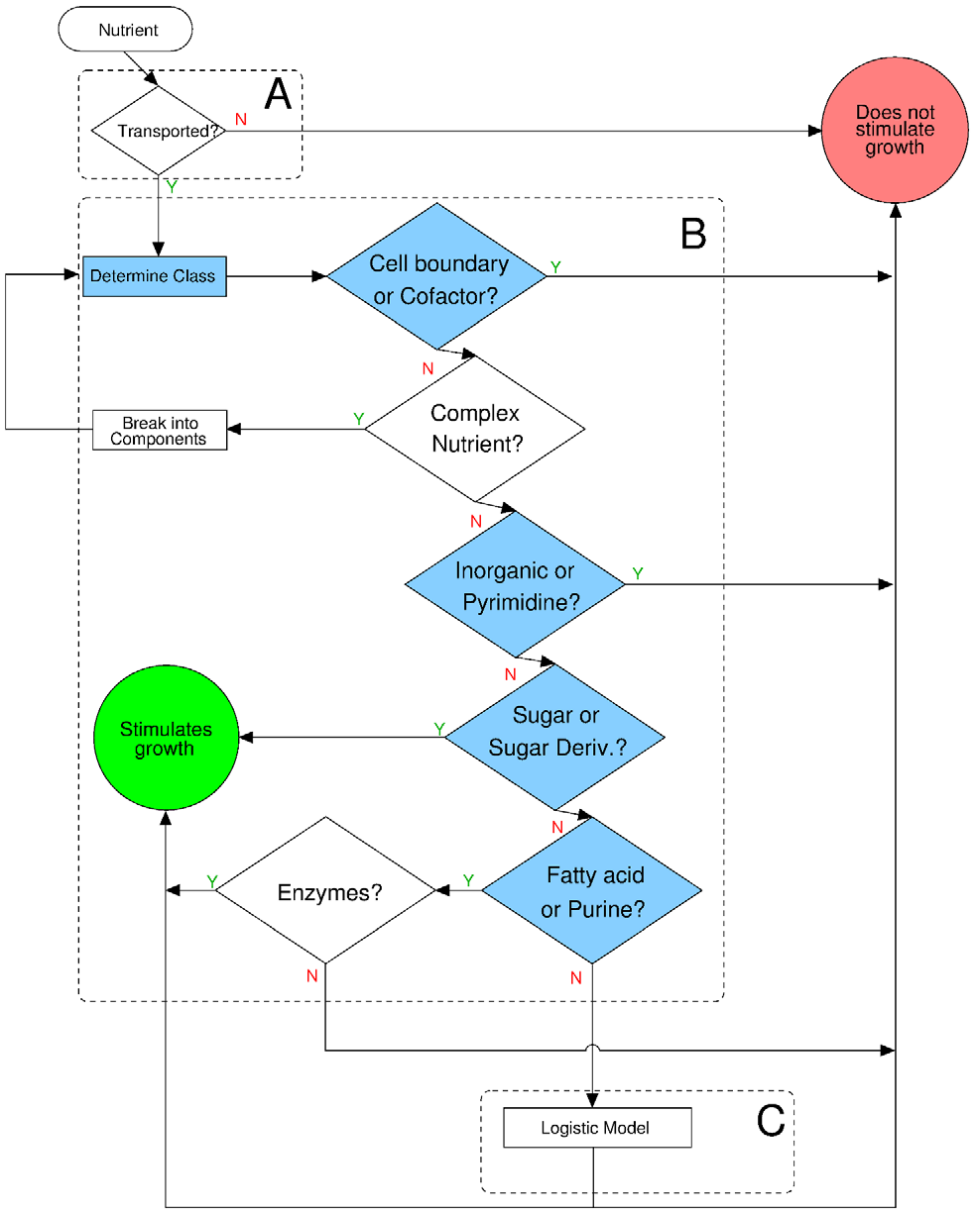
Determining whether a nutrient can or cannot be catabolized. **A**, To establish whether a given nutrient is a source of carbon for a given organism, we first need to determine whether the nutrient can be transported into the cell from the extracellular medium. **B**, For some nutrients, we can predict if a it does or does not contribute to growth just from knowing to which class it belongs. Complex nutrients are broken down into simple nutrients. See the main text for the description of the enzymes that catabolize fatty acids and purines. **C**, Any nutrient that is not classified into the nutrient classes in **B** is classified as G or NG using the logistic model described in the text and Methods.

The first step is to build a model that predicts whether an individual nutrient 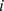 can be used as a source of carbon by species 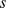 (see Fig. 1). If it can, we say that 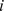 belongs to the group of nutrients 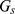 that contribute to growth in 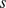; otherwise we say that 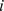 belongs to the group of nutrients 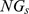 that do not contribute to growth in species 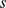. We use flux balance analysis on the species in the training set to empirically determine which nutrients belong to 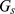 and which ones to 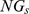 (see Materials and Methods). We then use these data to build the model. In this section we describe the model (which we summarize in Fig. 2) and validate it with the species in the test set.

#### Uptake

To establish whether a given nutrient is a source of carbon for a given organism, we first need to determine whether the nutrient can be transported into the cell from the extracellular medium (Fig. 2A). This question can be currently answered using bioinformatics tools. Recent studies have shed light on the function and orthology of families of microbial transporters for several nutrient classes [36]–[39]. Therefore, a search within a genome for orthologs of transporters provides a plausible answer as to whether a given nutrient is actively transported by a particular species.

**Figure 3:**
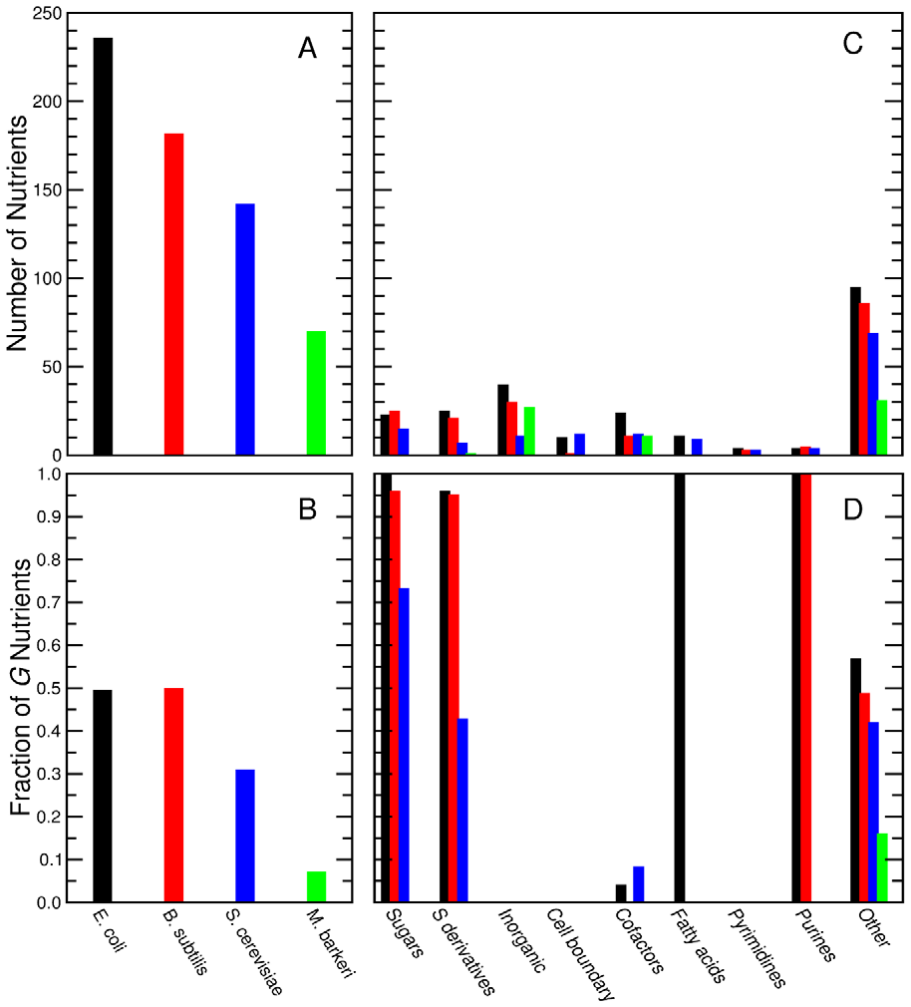
Nutrients uptaken and that stimulate growth in the presence of minimal media for the organisms in the training set. **A**, *E. coli* and *B*. subtilis have the largest number of uptaken nutrients whereas *M. barkeri* has the fewest. This reflects the current understanding of *M. barkeri* as a specialized methanogen [68]. **B**, *E. coli* and *B. subtilis* are able to catabolize half of the nutrients they uptake whereas *M. barkeri* can catabolize less than 10% of the nutrients it uptakes. **C**, Number of uptaken nutrients by nutrient class. Within each class, the four organisms uptake approximately the same number of nutrients. Exceptions are *M. barkeri*—which does not uptake neither Fatty acids nor Sugars, and only one Sugar derivative—and *B. subtilis*—which is not assumed to uptake Fatty acids in the *in silico* reconstruction. **D**, Fraction of uptaken nutrients that stimulate growth by nutrient class. There is a consistent pattern of growth stimulation across all four species for six nutrient classes: Sugars, Sugar derivatives, and Purines are catabolized whilst Inorganic compounds, Pyrimidines, Cofactors, and compounds involved in the formation of the cell membrane or cell wall (Cell boundary class) are not catabolized.

The number of nutrients that are actively transported by a species varies greatly with nutrient class (Fig. 3B and Methods for a detailed explanation of nutrient classes). For example, *M. barkeri* does not uptake nutrients that we classify as sugars, sugar derivatives, cell boundary, fatty acids, purines, or pyrimidines. However, experiments with *M. barkeri* may have deliberately been focused on small organic compounds, so one may question the range of nutrients that *M. barkeri* is *truly* capable of uptaking. Note that we will have to ask this question for every new *in silico* organism, an infeasible task given the large number of experiments that one would need to perform.

Fatty acids are only actively transported by two of the species we analyze, but the absence of transporters in the other species does not mean that they cannot allow the diffusion of fatty acids through the cell membrane [40]. In the following, we assume that all species can uptake fatty acids in this manner.

#### Nutrient classes

Next, we investigate whether entire classes of nutrients (defined by chemical structure and cellular function) can be labeled 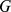 or 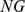 on the basis of the fraction of 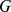 and 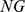 observations found for the training set of organisms. If this is the case, we would then be able to predict if a single nutrient is 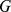 or 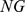 just from knowing to which class it belongs (Fig. 2B).

Based on the predicted biological function of the nutrients, we identify two classes whose nutrients are consistently found *not* to be sources of carbon: cell boundary nutrients and cofactors. Nutrients in the cell boundary class form the cell wall or the cell membrane and are composed of distinct metabolites that could, in principle, serve as carbon sources on their own. For example, lipid A contains several fatty acid chains that can be catabolized in *E. coli*. However, as in this particular example, these complex nutrients do not have enzymes available to decompose them, and they are instead used directly in the formation of the cell boundary. Cofactors have several metabolic functions (they serve as precursors to several biomass components, facilitate or act as carrier molecules in many reactions, act as transporters of important inorganic elements such as iron), but nearly none of them is catabolized by the species we study. Functional classes highlight that if a compound has a function in the cell other than being a source of carbon, then there will be selective pressure against the occurrence of enzymes that breakdown these nutrients.

When considering the chemical structure of nutrients, we find four nutrient classes whose components are consistent in whether they were sources of carbon or not: inorganic compounds, pyrimidines, sugars, and sugar derivatives. We label inorganic compounds and pyrimidines as 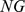 since none of these nutrients acts as a source of organic carbon on its own for any of the species in the training set (Table S6b in Text S1). We label all sugars and sugar derivatives as 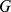, a classification that is accurate 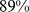 of the times. This level of accuracy is expected since these are typically the major sources of carbon for many species (Tables S6d & e in Text S1). There are 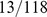 observations where sugars or sugar derivatives are 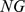, despite the organisms having transporters available for their uptake. This means that the organisms have no enzymes that can catabolize these sugars and their derivatives, and it is likely that they are uptaken because of the lack of specificity of some sugar transporters [41], [42]. One notable exception is inositol which can be involved in signaling processes [43] or in pathogenesis [44]; we surmise that, for this reason, some species may select against the presence of enzymes that allow inositol to be catabolized.

 We find mixed results outside of the four structural classes discussed above. Purines (Table S6a in Text S1) are 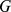 in *E. coli* and *B. subtilis*, but 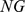 in the other species in the training set. In *E. coli* and *B. subtilis*, purines are catabolized via the degradation of urate into glyoxylate; we find a well-characterized enzyme in this pathway that is present in these species, but is not present in the other two: 5-hydoxyisourate hydrolase (EC: 3.5.2.17). Therefore, if an organism contains genes encoding for this enzyme we label all purines uptaken by that organism as 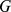; otherwise, we label them as 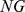.

Because we assume that fatty acids can always be uptaken by a microbe, we need to be able to predict whether a given fatty acid is 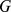 or 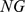 on the basis of the enzymes available in the organism's genome. The catabolism of fatty acids occurs through a self-contained pathway that produces acetyl-Coenzyme A (CoA) by way of 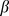-oxidation [45]. A round of reactions in the 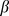-oxidation process may depend on whether the fatty acids are saturated or not, but the final two reactions are always the same: i) the oxidation of L-3-hydroxyacyl-CoA to 3-Ketoacyl-CoA (EC: 1.1.1.35) and ii) the cleavage of 3-Ketoacyl-CoA with another CoA molecule to produce acetyl-CoA (EC: 2.3.1.16). If an organism contains genes encoding for the enzymes catalyzing both of these reactions, we label all fatty acids uptaken by that organism 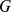; otherwise, we label them 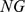.

#### Logistic model for other nutrients

The remaining nutrients cannot be globally classified as 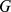 or 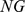 based on their structure or function. Therefore, we investigate their catabolic potential on the basis of the metabolic pathways in which they participate (as defined by KEGG [33]–[35]; see Methods).

We use logistic regression [46] to estimate the probability 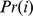 that nutrient 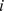 is a source of carbon, that is 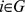, given a model 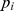


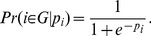
(1)We consider the following linear model for 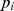:
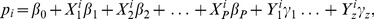
(2)where 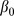 is related to the probability that nutrient 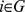 if it is not in any of the pathways in the model, 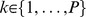 runs over the pathways included in the model, 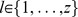 runs over the pairs of pathways included in the model, 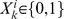 indicates whether a nutrient belongs to pathway 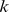, 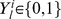 indicates whether a nutrient belongs to a given pair of pathways, and 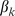 and 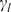 are interpreted as the change in the probability that nutrient 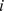 can be catabolized by belonging to pathway 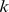 or to the pair of pathways 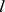, respectively. We performed the logistic regression using R (version 2.10.1 [47]).

**Figure 4:**
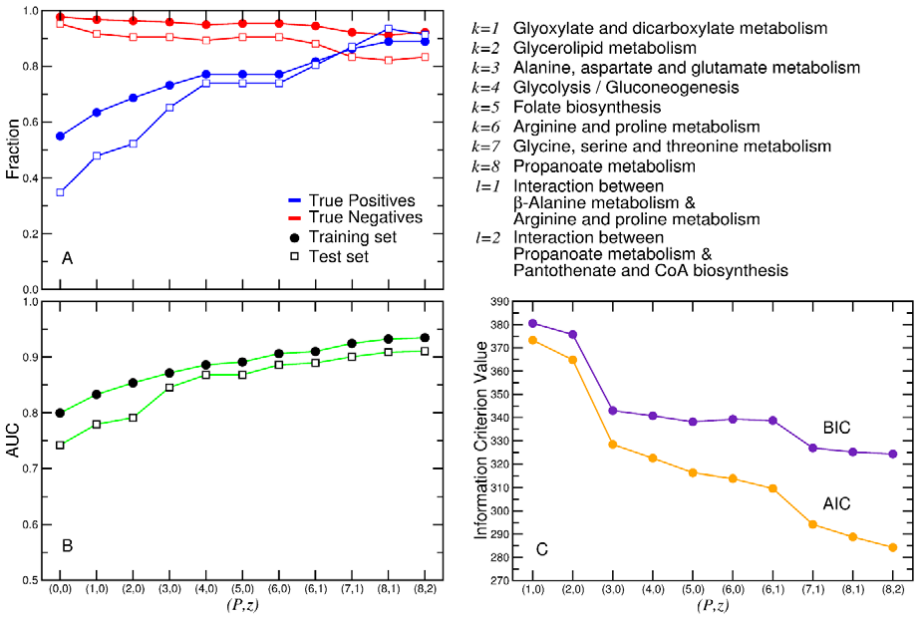
Model selection. We consider logistic models with different number of pathways *P* and of pairs of pathways *z* (see text and Methods). **A**, Model accuracy. We calculate the true positive (*TP*) and true negative (*TN*) rates for the different models. *TP* reflects whether the model correctly predicts *G* nutrients, whilst the *TN* reflects whether the model correctly predicts *NG* nutrients. **B**, Area under the ROC curve (*AUC*) for the 10 models. The higher the *AUC*, the better the model is at separating *G* nutrients from *NG* nutrients. **C**, Akaike information criterion (AIC) and Bayesian information criterion (BIC) of the 10 models. The lower the information criterion, the more parsimonious the model. We could not identify any additional pathways and/or pathway pairs that improved the AIC and BIC of the model with *P* = 8, *z* = 2 (pathways and pathway pairs are listed in the upper right panel of the figure). In the case of *TP*, *TN*, and *AUC*, we apply our complete model including both nutrient classes and KEGG pathways to the training set of organisms, and to the test set of organisms (see text and Methods). When *P* = 0, *z* = 0, there are more *NG* nutrients than *G* nutrients that are not included in a nutrient class, therefore all of these nutrients are considered *i*∈*NG*; hence, the initially low *TN* rate. When *P*≥4, the *TP* in the test set is similar to the *TP* in the training set. This means that our model is successful at identifying *G* nutrients. However, the *TN* for the test set is slightly lower than the TN for the training set. This occurs because there are more *NG* nutrients in the test set that are also found in the Sugar and Sugar derivative classes, or in *G* pathways in the linear model, which we could not account for because of the small sample size of the training set. The difference between the *TN* rates of the two test sets has an impact on the overall accuracy of the model for the training and test sets.

We use the logistic model above to uncover a small set of pathways that has the greatest predictive power for the largest number of nutrients (Fig. 2C). We start with an initial null model of zero pathways, and build increasingly sophisticated models by sequentially adding linear terms corresponding to the pathways that provide the greatest increase in prediction accuracy (Fig. 4).

As our first strategy, we add terms by selecting a pathway with both a high *number* of 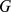 nutrients and a large *fraction* of 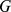 nutrients. Many pathways with only one or two 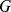 nutrients have 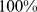 of 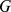 nutrients, but will not achieve our goal of covering the greatest number of nutrients. For 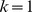 (only one term in the model), we select “Glyoxylate and dicarboxylate metabolism,” which has thirteen 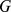 nutrients and only two 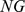 nutrients. When we consider which pathway to add next, we only consider the nutrients that are not already accounted for in the model, which is important because there is a significant overlap between pathways. For example, when determining which pathway to select for 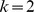, we note that after including “Glyoxylate and dicarboxylate metabolism”, we *exclude* fifteen nutrients from the rest of the pathways (Table S7 and Fig. S1 in Text S1). This has the additional effect of removing two pathways from consideration.

As our second strategy, we consider pathways with a high fraction of 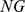 nutrients. By doing so, we build a model that can predict 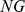 nutrients as well as 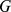 nutrients. There are several pathways that have a high fraction of 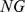 nutrients, but we find that only the inclusion of “Folate biosynthesis” significantly improves the model.

As a third strategy, we add pairwise interaction terms to the model. These terms reduce the number of false positives due to all nutrients in a pathway with high fraction of 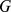 nutrients being predicted to be catabolized. We find that there are several pathways that contain 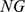 nutrients also found in the 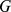 pathways in the model. These pathways do not necessarily have a high fraction of 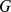 or 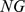 nutrients, but they enable us to pick out nutrients that are false positives in the pathways classified as 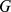.

Our analysis identifies a logistic model with 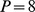 pathways, and 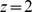 pathway interactions (Table S8 in Text S1). The small number of pathways could be a function of the small number of species we are exploring. However, we find that 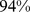 of the thousand or so microbes listed in KEGG have the pathways included in our model, suggesting that, despite its simplicity, our model is likely to be applicable with similar accuracy to other species.

#### Model validation

**Figure 5:**
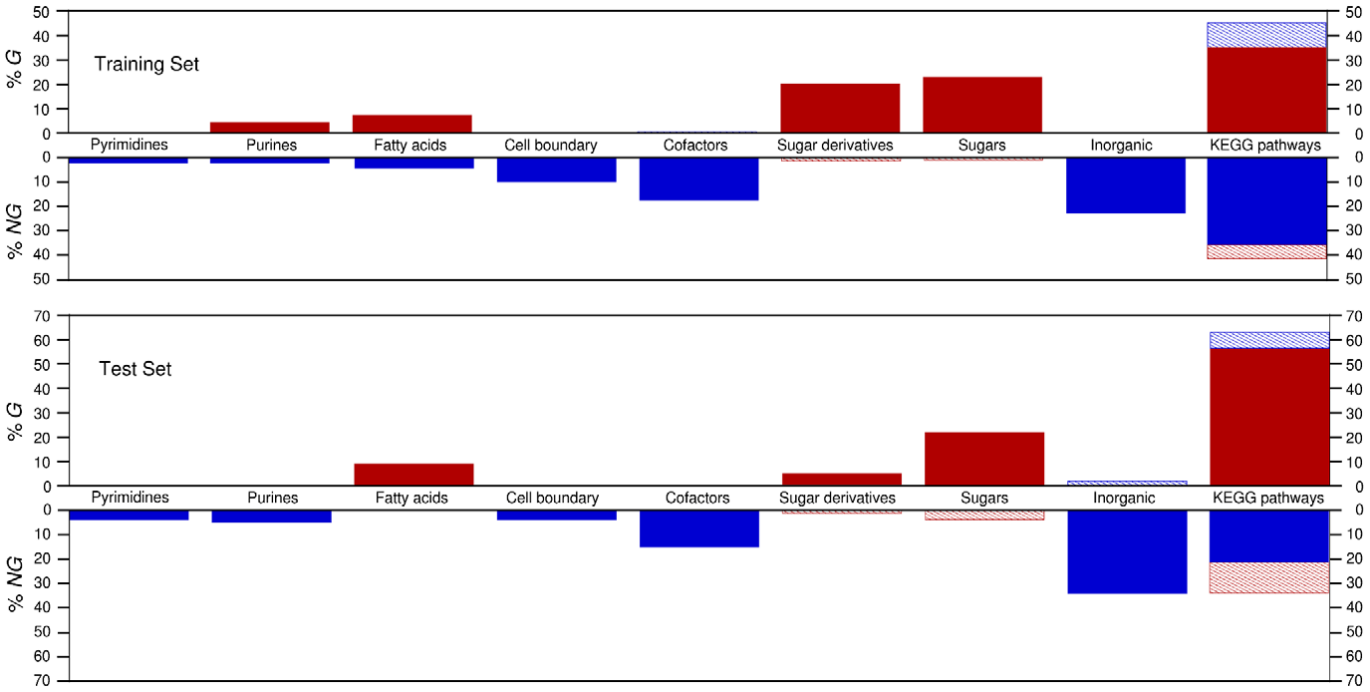
Breakdown of true positives and true negatives in training and test sets. Solid red indicates true positives. Solid blue indicates true negatives. Hashed red indicates false positives. Hashed blue indicates false negatives. If our model was 100% accurate, the solid red bars would add up to 100%, as would the solid blue bars. It is visually apparent that the majority of false positives and false negatives are due to misclassification using the KEGG pathways.

Our final model for catabolic potential integrates 7 nutrient classes and 10 pathways. Using our training set of species, we find that the true positive rate is 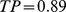 and the true negative rate is 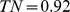 (Fig. 4 and Fig. S2 in Text S1). To validate the model beyond the training set, we test its predictions on the set of test organisms described above. We find that the 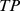 rate is consistent between the training set and the test set of nutrients, whereas the 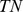 rate drops slightly (Fig. 5). This drop is mostly accounted for by nutrients that are 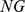 in the test set, but that are found in the 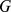 pathways of the training set and are not corrected by the interaction terms in our model, thus providing an avenue for improving the model.

### Biomass yield: What is the biomass yield of a given nutrient for a specific microbe?

**Figure 6:**
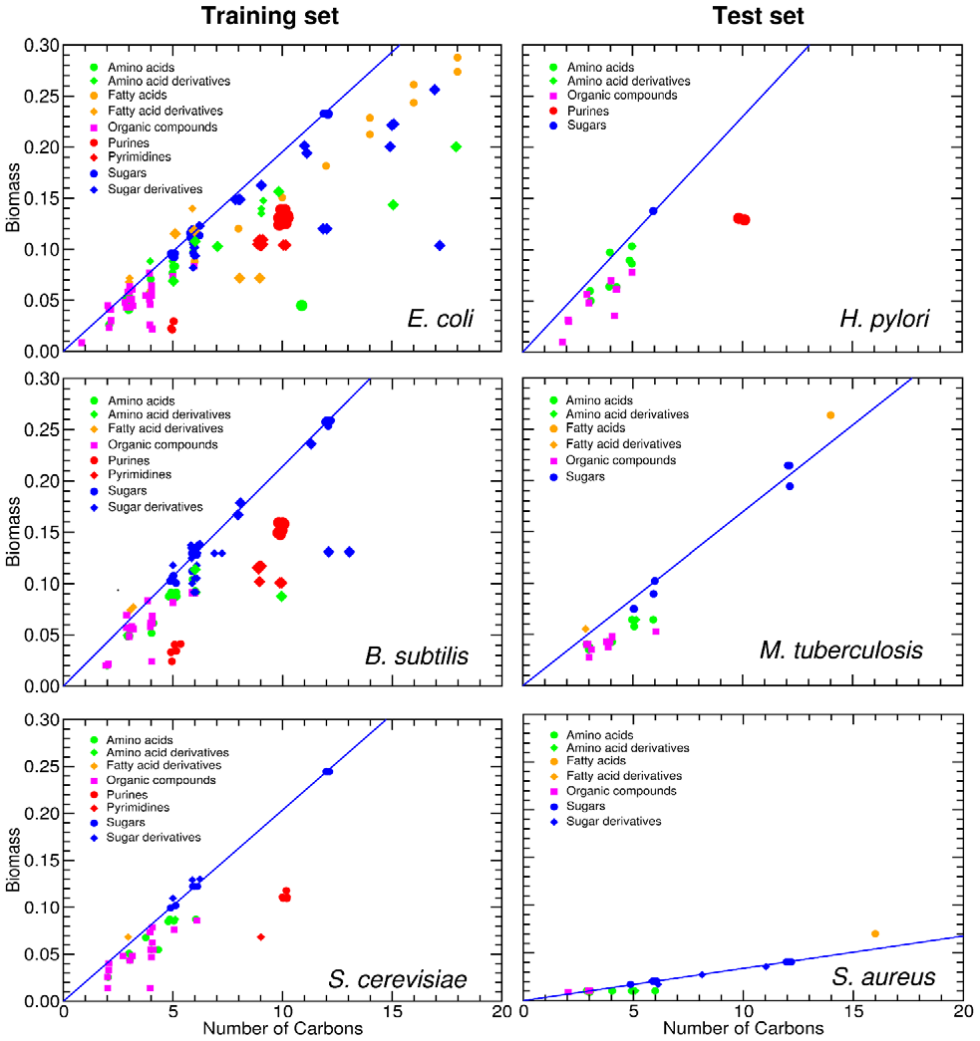
Biomass production is related to the number of carbons in a nutrient. We show the optimized biomass production of each species on *G* nutrients, for species in the training set (left) and the test set (right). For all species there is a positive correlation between biomass production and the number of carbons in the nutrient. The blue line represents 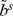 (see text) for all the sugars uptaken by species *s*. *S. aureus* exhibits a reduced biomass production; the biomass defined in the *in silico* organisms demands approximately ten times more moles relative to the other species. In all the plots, the position of the nutrients on the X axis is slightly staggered so that all data points are visible. Note that the symbols for the complex nutrients are enlarged.

The next step is to determine for those nutrients that produce growth what is the maximal production of biomass when that nutrient is the only source of carbon. In fact, if a nutrient is the carbon-limiting source in a given medium, the biomass yield of the nutrient must be related to the number of carbons in the nutrient. In Fig. 6, we display the *in silico* biomass production 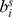 and the biomass yield 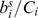 of all nutrients as a function of the number 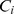 of carbon atoms they contain, for the species in the training and test sets (we do not consider *M. barkeri* in our model for biomass yield because *M. barkeri* can only grow anaerobically, and this has a significant effect on the energy used to polymerize the proteins and nucleic acids in the biomass).

**Figure 7:**
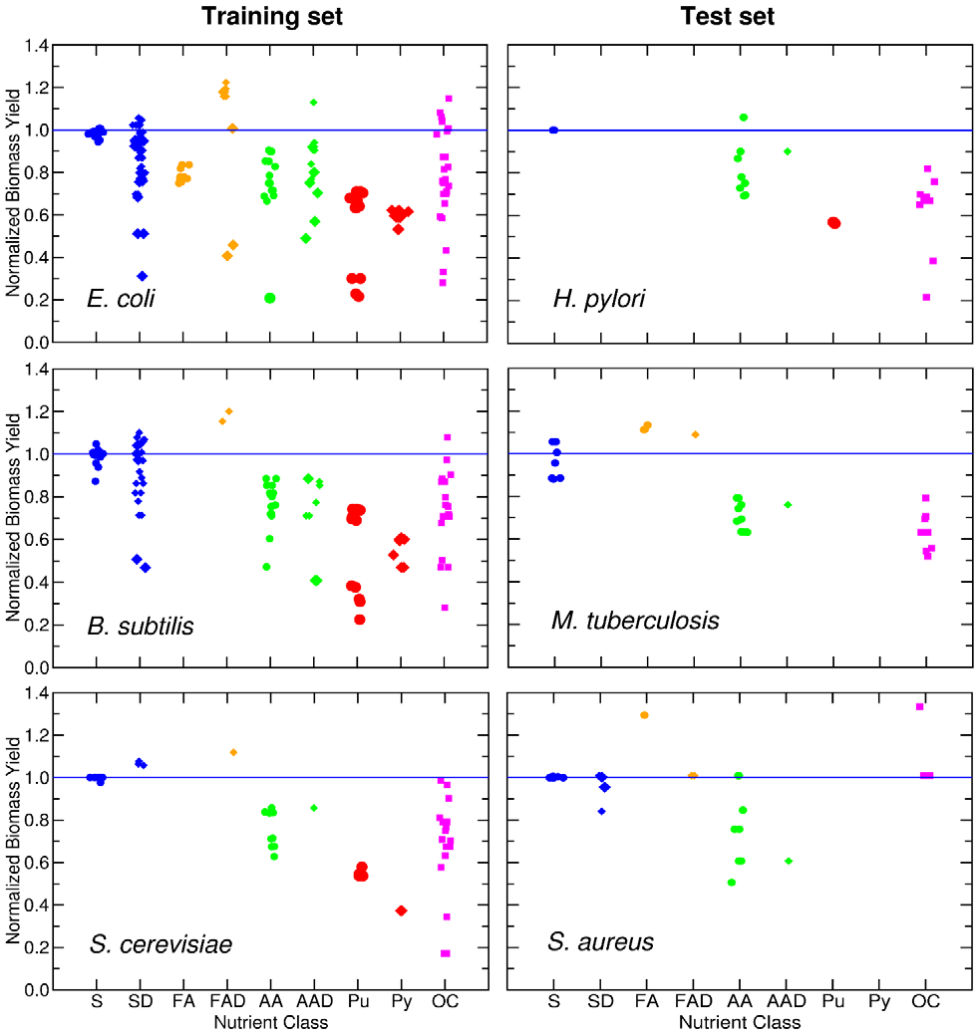
Normalized biomass yield for nutrient classes. The panels show the normalized biomass yield 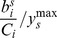 of *G* nutrients for species in the training set (left) and in the test set (right). Nutrients are grouped by their nutrient class (with positions in the X axis staggered so as to allow one to see all of them). The blue line represents 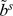 for all the sugars uptaken by species *s*. The symbols for complex nutrients are enlarged.

**Figure 8:**
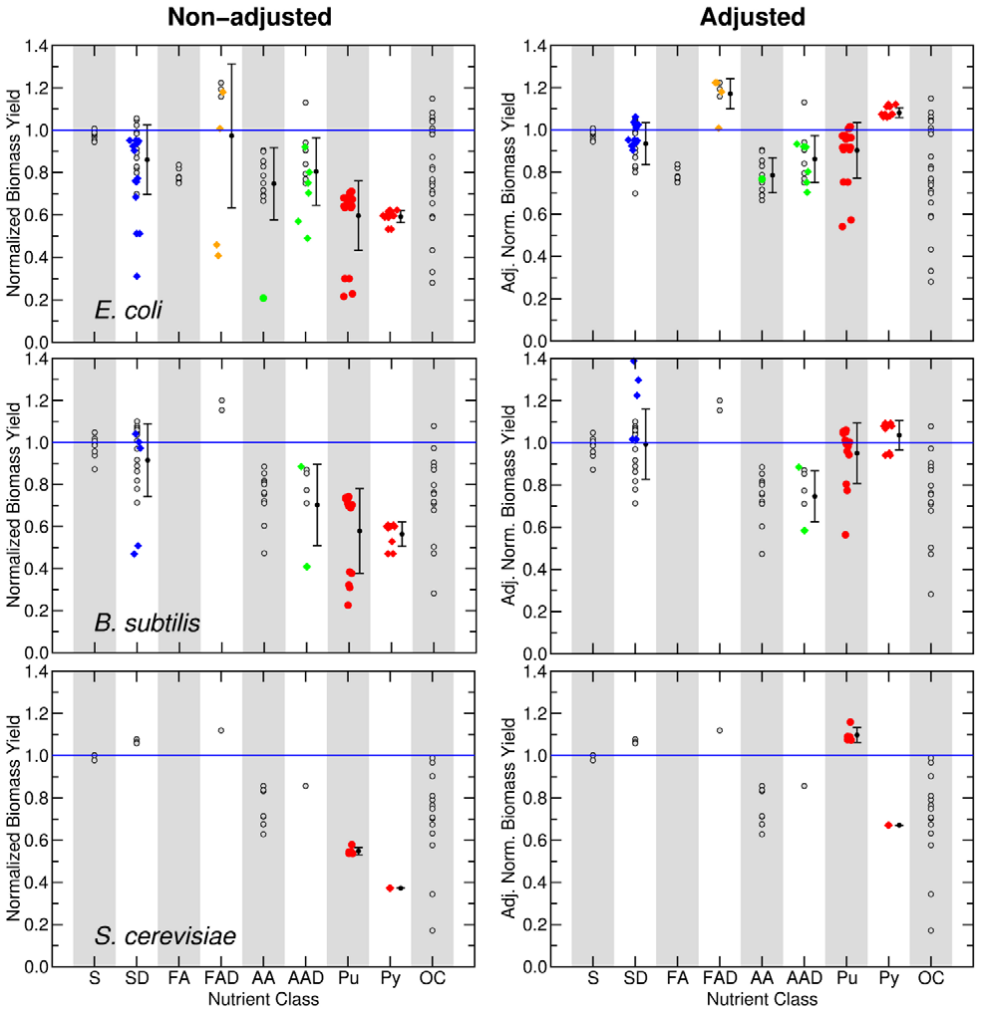
Adjusting for the effective number of carbons in complex nutrients and purines in the training set. We show the normalized biomass yield (see Fig. 7) for purines and complex nutrients (full colored symbols) for species in the training set. In the left column, we show the normalized biomass yield considering the number of carbons in each nutrient *C_i_*. In the right column, we show the normalized biomass yield using the effective number of carbons 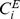 (see Text and Methods). Additionally, for each nutrient class that contains these nutrients, we show the mean and variance.

It is visually apparent from Fig. 6 that there is a strong correlation between the number of carbons in a nutrient and the biomass production. We thus model the biomass production induced by nutrient 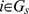 as
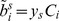
(3)where 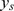 is the (nutrient-independent) average biomass yield of the nutrients in 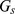


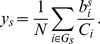
 The data suggests the existence of an upper bound for the biomass yield (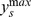) as a function of the number of carbons (blue line in Figs. 6, 7, and 8). The nutrients frequently found at or close to this upper bound are sugars, alcohols, and other compounds with hydroxyl groups. Many of the hydroxyl groups on these compounds are typically oxidized in order to reduce NAD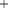 to NADH, which is an important source of energy, and will in turn increase the biomass yield. For simplicity, we obtain 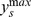 as the average biomass yield for the sugars uptaken by an organism. The average nutrient has 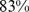 of the efficiency of these high-efficiency sugars, but nutrients vary wildly in their yield (Fig. 7). We consider one main factor that contributes to this variation in biomass yield: the number 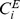 of carbons in a nutrient that are *effectively* catabolized.

#### Effective number of carbons

We find two sets of nutrients for which the number of carbons that is effectively catabolized is not the actual number of carbons available in the nutrient 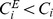: complex nutrients and purines.

Very often, catabolism of complex nutrients starts by breaking them down into a number of simpler nutrients, which are then catabolized according to their respective pathways. Tryptophan, lipids and nucleotides are examples of such complex nutrients: tryptophan is broken down into three separate metabolites (pyruvate, indole, and ammonia); lipids are broken into fatty acids, glycerol and a functional group such as choline; nucleotides are broken into a phosphorylated ribose and a nucleobase. Each of these simple compounds can act as nutrients on their own.

Complex nutrients display some of the largest variation in biomass yield, as highlighted in Fig. 8. This can be explained by the fact that not all of the constituent simple nutrients can be catabolized, that is, not all of the carbons in a complex nutrient can be used to produce biomass. Therefore, for each complex nutrient 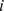 we estimate 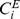 as follows. We break down the nutrient into its respective simple nutrients. Then, we find whether each simple nutrient 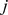 can be catabolized; 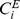 is the sum of 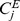 for each simple nutrient 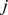 that can be catabolized
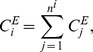
where 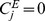 if 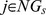 and 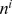 is the number of simple nutrients that compose the complex nutrient 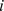. For example, consider the nucleotide thymidine for which 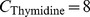. Thymidine is comprised of two simple nutrients, the phosphorylated ribose (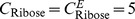), and thymine (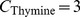, but 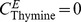 because 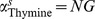). Therefore 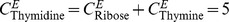.

An additional consideration is the fact that some nutrients “leak” carbons during catabolism. Purines are an example of this; they are reduced to glyoxylate, but in doing so, they “leak” three carbons as carbon dioxide and urea, which cannot be catabolized for the carbon. Therefore 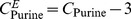.

Figure 8 displays how the biomass yield of complex nutrients changes when we adjust for the effective number of catabolizable carbons. Tryptophan, for example, is now seen to be catabolized as efficiently as other amino acids. Many purines and sugar derivatives only contained sugars as catabolizable simple nutrients, and therefore their efficiency becomes comparable with that observed for nutrients in the sugars class. Adenine and guanine still remain inefficient (the two red dots towards the bottom of the purines class), but as their catabolic end product is glyoxylate, the result is comparable with a glyoxylate derivative highlighted in the organic acids class.

For some classes, such as amino acids and sugar derivatives, the variance of the biomass yield for nutrients within the classes is also reduced. This reduction supports the use of the effective number of carbons as the predictor of biomass yield of a single nutrient. We thus modify Eq. (3) accordingly:
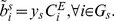
(4)Note that species in the test set take up very few complex nutrients and no purines; we therefore do not show the results of using Eq. (4) for these species.

### Complex media: Is biomass yield of a nutrient affected by the presence of other nutrients in the medium?

Finally, we want to model the maximal biomass production when there is more than a single source of carbon present in the medium, or in other words when organisms grow in a complex medium. We consider complex media in which nutrients are restricted to five classes: sugars, fatty acids, amino acids, purines, and pyrimidines, partly because they are commonly used in growth rate/biomass yield experiments, and partly because it simplifies the analysis of the results. In addition, complex purines and pyrimidines are a mixture of sugars and nucleobases, and as we are already considering sugars, we only use the simple nucleobases.

The simplest plausible model for the contribution of nutrients to the biomass production is one in which each nutrient 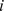 has an *independent contribution* to the biomass production. For each species 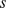, we calculate the biomass production 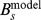 on a complex medium containing 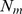 nutrients using
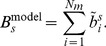
(5)We estimate 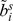 for a nutrient from class 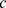 using the average yield for that class in the organisms in the training set
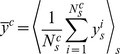
(6)where 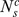 is the number of nutrients of class 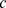 that are taken up by species 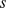, and 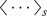 represents the average over species in the training set. For tryptophan and for purines, we use the effective number of carbons when calculating yield, as described previously.

**Figure 9:**
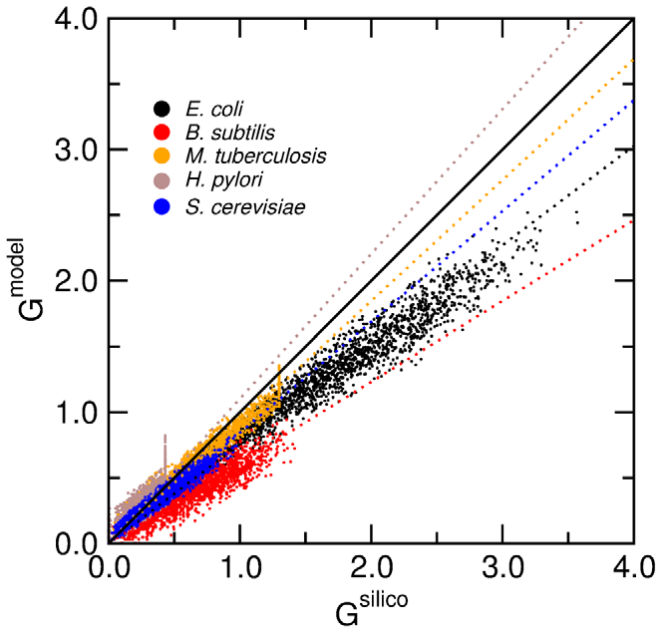
Validation of the model for biomass production on complex media. We show, for 3000 randomly generated complex media containing sugars, fatty acids, bases, and amino acids (see Methods), the prediction for the biomass production as a function of the actual *in silico* growth. We show the results for *E. coli, B. subtilis, S. cerevisiae, H. pylori*, and *M. tuberculosis*. The dashed lines represent a regression of the predicted biomass production versus experimental *in silico* production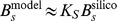. We obtain: *K* = 0.76 for *E. coli, K* = 0.62 for *B. subtilis*, *K* = 0.84 for *S. cerevisiae*, *K* = 1.10 for *H. pylori*, and *K* = 0.92 for *M. tuberculosis*.

To test this model, we randomly generate ensembles of complex media as described in the Data and Methods section. For each medium and for each species, we calculate 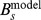; we use FBA to find the actual *in silico* biomass production of the organism on the complex media 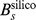. In Fig. 9 we show how 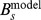 compares to 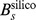 for each species, and for each of the 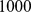 complex media we generate. In these comparisons we also include predictions for the species in the test set (with the exception of *S. aureus*, for whose reconstruction it is not clear what units were used for the biomass production).

We find a very strong correlation (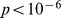 using the Spearman rank correlation coefficient) between the predictions of our model and *in silico* experimental results for training set species (*E. coli*, *B. subtilis*, and *S. cerevisiae*) and test set species (*H. pylori* and *M. tuberculosis*). This indicates that the model accurately captures which media will result in faster/slower production of biomass. For each species, however, the model systematically under or over-predicts growth. A regression of the predicted biomass production versus experimental *in silico* production indicates that 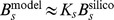, with: 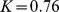 for *E. coli*, 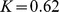 for *B. subtilis*, 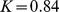 for *S. cerevisiae*, 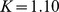 for *H. pylori*, and 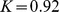 for *M. tuberculosis*.

The parameters used to make the predictions for the individual nutrients in the linear model were trained on three species. The linear model consistently under-predicted the *in silico* biomass production for these three species, and more so for media containing more nutrients. This is a strong indicator that the nutrients that are uptaken are used synergistically by the *in silico* organism to produce *more* biomass than expected, showcasing the effect of catabolic pathways being highly connected in the metabolic network. A more complete model for predicting biomass production in complex media will therefore need to take into account synergistic interactions among catabolic pathways.

### Predicting growth for organisms lacking a metabolic reconstruction

Our model sheds light on several questions related to the impact of nutrients on the biomass production of microbes. Our approach treats microbial metabolism as a “black box” that uses nutrients to reach optimal biomass production. Because the model does not take species-specific details into consideration, it is useful for generating predictions for any microbe, something that would be impossible with any existing modeling approach. To illustrate how one would proceed to extrapolate to “new” organisms and to show what kind of insights one could obtain, we generate predictions for four organisms that lack a metabolic reconstruction: *R. palustris* (a gram-negative bacterium), *L. monocytogenes* (a gram-positive bacterium), *D. discoideum* (an eukaryote), and *T. acidophilum* (an archaeon).

**Figure 10:**
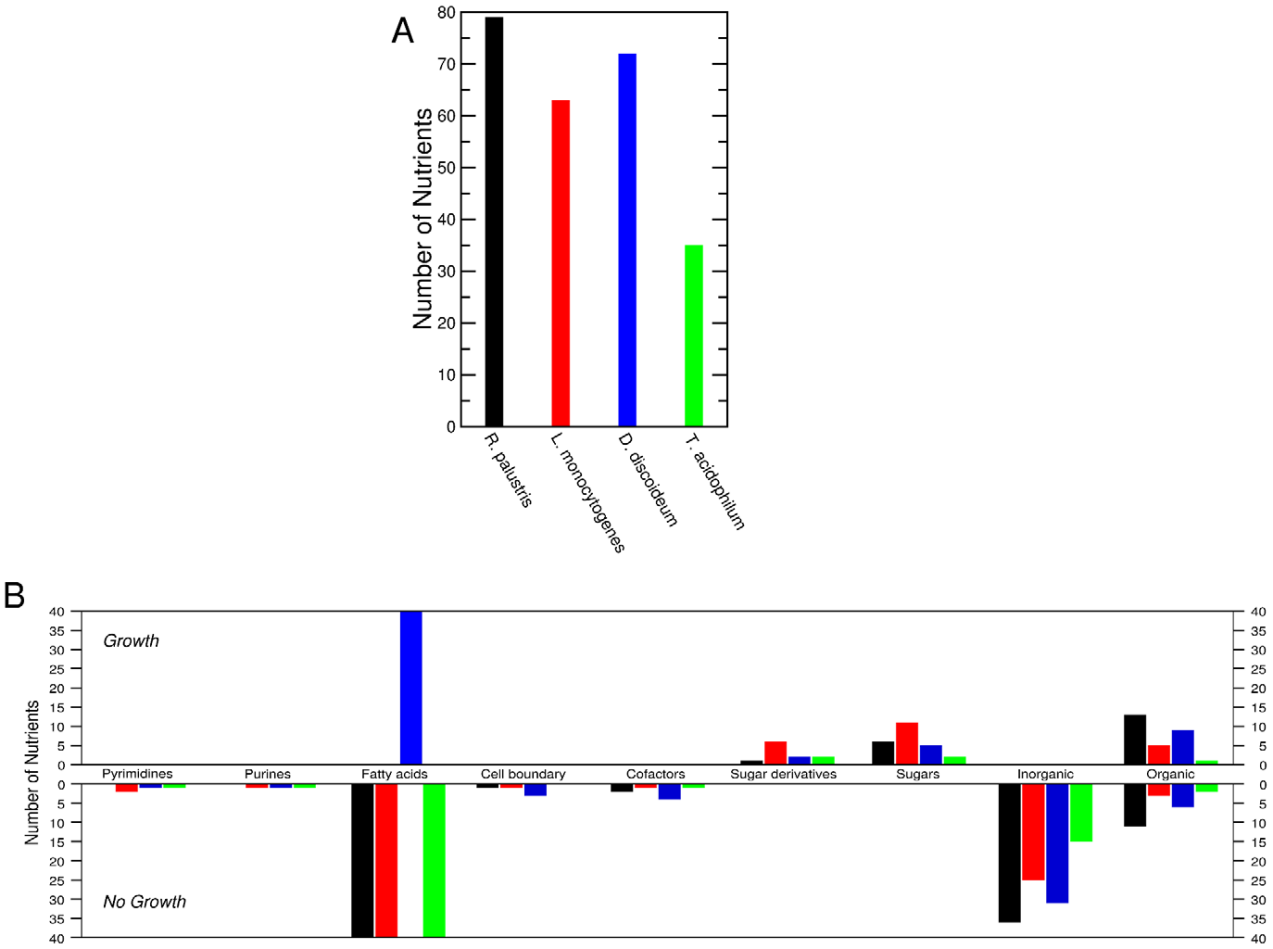
Predictions for four organisms lacking a metabolic reconstruction. **A**, The number of nutrients found to be uptaken by four organisms for which we lack a metabolic reconstruction: *Rhodopseudomonas palustris* (gram-negative bacterium), *Listeria monocytogenes* (gram-positive bacterium), *Dictyostelium discoideum* (eukaryote), and *Thermoplasma acidophilum* (archaeon). The nutrients were determined using predictions found in TransportDB (http://www.transportdb.org). **B**, Prediction of whether a nutrient is a source of carbon according to class. Bars in the top panel represent predictions of *G* nutrients, whereas bars in the bottom panel represent predictions of *NG* nutrients. None of the species had fatty acids listed as nutrients, but since fatty acids can be uptaken by diffusing through the cell membrane, we show here the predictions for fatty acids as well. The prediction for nutrients in the Organic compounds class are based on our logistic regression using the KEGG pathway terms. Thus some nutrients are predicted to be *G* while others are predicted to be *NG*.

We find that, overall, these species are predicted to take up fewer nutrients than the species in the test set (Fig. 10A). This is a consequence of the limited annotation that the authors of TransportDB could use for the predicted protein transporters. One example of this limitation is that for all four species, there were many transporters predicted to take up amino acids, but there was no indication of which amino acids the transporters were specific for. Therefore, for the sake of prediction, we consider that all four species uptake all twenty natural amino acids.

We then use the model of catabolic potential to predict whether each of these nutrients could be a source of carbon. In Fig. 10B we show the number of nutrients that belong to one of the nutrient classes previously described and whether these nutrients are 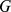 or 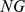. For the fatty acids, none of which were predicted to be uptaken by any of the four species, we examined the enzymes available and found that only *D. discoideum* contains the enzymes for 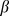-oxidation, and could therefore catabolize fatty acids.

**Figure 11:**
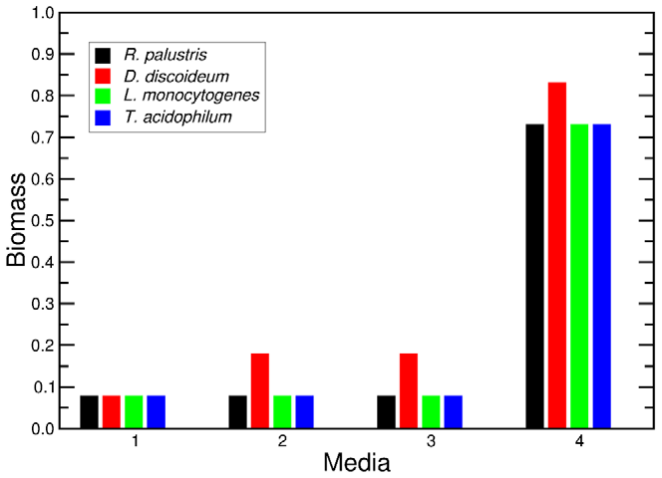
Predictions of biomass production for four organisms lacking a metabolic reconstruction. The predictions are made for the biomass production of *R. palustris*, *D. discoideum*, *T. acidophilum*, and *L. monocytogenes*. For the predictions of biomass production, we use four different complex media containing: 1) Glucose 2) Glucose and hexanoic acid 3) Glucose, hexanoic acid, guanine, adenine, cytosine, and thymine. 4) Glucose, hexanoic acid, guanine, adenine, cytosine, thymine, and the 20 natural amino acids (see Methods). There is no difference in the prediction for biomass production between complex media 2 and 3 because none of the species shown here can catabolize nucleobases. The large number of carbons available in the 20 natural amino acids are responsible for the increase in biomass production predicted for complex medium 4.

Finally, we combine our models of biomass yield of 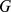 nutrients, and of biomass production on complex media (Fig. 11). We choose four complex media which contain a different number of the same nutrients we used for the randomly-generated complex media, namely: sugars, fatty acids, bases, and amino acids. The four media contain: 1) glucose; 2) glucose and hexanoic acid; 3) glucose, hexanoic acid, adenine, guanosine, cytosine, and thymine; 4) glucose, hexanoic acid, adenine, guanosine, cytosine, and thymine and the twenty natural amino acids. We estimate the biomass production in the same manner that we described earlier for the randomly-generated complex media. We find that the biggest influence on the biomass production is the number of carbons available in the nutrients present in the medium; because we are now adding 20 amino acids to medium 4, the biomass production increases almost 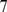-fold, on average, in that case.

Note that these predictions for biomass production are based on the biomass yield per carbon available in the nutrients present in the medium. Biomass yield is a time independent quantity that cannot be directly associated to growth rate. However, because biomass yield gives an upper limit for growth rate [48], our model can be used as a baseline for researchers to explore and model growth rates.

## Discussion

Microbes use nutrients found in their environments to grow. Understanding and developing quantitative models of this process is of fundamental importance in cell biology, physiology, medicine, evolution, synthetic biology and bioengineering, not to mention of practical importance to those that need to grow microbes in laboratories. Given the diversity of the microbial world and the number of combinations of nutrients available in their environments, this seems too difficult a problem. In this study, we focused on how microbes might catabolize nutrients to obtain carbon for biomass production.

Our model comprises three levels, with each level building up on the results from the previous one. The first level concerns whether a nutrient *will* be catabolized. The second level concerns whether all of the carbon in a catabolized nutrient is available for biomass production. The final level incorporates the biomass yield for selected classes of nutrients and enables us to make a prediction on the biomass production of a microbe in a complex medium. To validate our approach, we compare the predictions of the complete model with *in silico* predictions of growth in complex media of species that are not part of the training set. Our results on these species are excellent predictions of which media will produce more/less growth.

Finally, we looked at the biomass yield of sugars, amino acids, purines, pyrimidines, and fatty acids. We found little variation in the yield of these nutrients amongst different species, and were able to postulate a model for the biomass production of a microbe on a complex medium containing any number of these important nutrients. All of these nutrients have separate catabolic pathways, with the exception of some groups of amino acids which share a catabolic pathway. The fact that the *in silico* microbial biomass production was 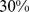 more than our model predicts indicates that each of these catabolic pathways can work together synergistically to improve the biomass production of the microbe.

The ability of our model to predict biomass production on complex media must be balanced by an understanding of its limitations. First, we model microbial metabolism as a black box, and therefore we do not account for absent pathways that biosynthesize some biomass components. The absence of such pathways would require that the corresponding biomass components be made available in the medium used, but our model cannot be used to predict which of these are indeed required. This means we cannot use the model in its current form to predict the minimal medium that is needed for a microbe to grow.

Second, microbial species are sometimes identified by the nutrients they take up and excrete. The ability of a microbe to transport such compounds in and out of the cell is largely dependent on the specific protein transporters available. A separate body of work exists, largely in the form of TransportDB [39], and TCDB [49], [50], which will enable the researchers to predict the proteins that transport specific nutrients. We incorporate such predictions of nutrient transport into our model to make predictions for specific species that lack a metabolic reconstruction. Importantly, while knowledge of transporters can enable us to predict which nutrients in the medium can be taken up, we are currently unable to predict which nutrients are excreted as that would in principle require knowledge of the full metabolic network.

Third, the stoichiometric method we describe for generating the biomass data cannot be used to predict the growth rate of a microbe because kinetic information is not included. However, our model provides a baseline to which one can add kinetic information. For example, the rate-limiting step in poor growth media is likely to be the rate at which a microbe takes up nutrients. Therefore one can use the kinetics of nutrient transport with our prediction of biomass yield to predict growth rate.

All this notwithstanding, we believe that our approach and models open the door to significant advances in the quantitative modeling of microbial metabolism, and eventually of the metabolism of more complex organisms. In particular, our models could be extended to consider whether a nutrient acts, not only as a source of carbon, but also as a source of nitrogen or energy, or directly as a component of the biomass. Our models could also be extended to include more detailed information about pathways, or to consider functional metabolic modules [51]–[53] instead of pathways.

## Methods

### Data

#### *In silico* organisms

We first consider a training set of four species for which there are high quality metabolic reconstructions available, and which cover a wide range of microbial phylogeny: a gram-negative bacterium (*E. coli* [8]), a gram-positive bacterium (*B.subtilis* [9]), an eukaryote (*Saccharomyces cerevisiae* [21]), and an archaeon (*Methanosarcina barkeri* [24]). We validate our model on a set of test species: *Helicobacter pylori*, a gram-negative bacterium [20], *Staphylococcus aureus*, a gram-positive bacterium [22], and *Mycobacterium tuberculosis*, an acid-fast gram-positive bacterium [23]. We download these organisms from http://gcrg.ucsd.edu/ in SBML format (accessed 03/27/2009). There are 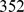 distinct nutrients listed for the metabolic reconstructions of the four *in silico* organisms in the training set. According to the reconstructions, out of these 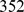 nutrients only 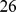 are uptaken by all four species, a fact that illustrates the diversity of microbial metabolism.

We use the available reconstructions and the literature to define a fully minimal medium for each *in silico* organism [54]–[57]. The minimal medium does not enable the organism to produce biomass. However, the addition of a carbon source is enough to enable growth. We do not consider components of a minimal medium for which an uptake reaction is not present in the reconstruction. We describe in detail the minimal media for the seven organisms in our training and test sets in Table S1 in Text S1.

#### Organisms in prediction set

We generated predictions of biomass production on four species for which we lack a metabolic reconstruction, and which have the same phylogenetic breadth as the training set: *Rhodopseudomonas palustris* (a gram-negative bacterium), *Listeria monocytogenes* (a gram-positive bacterium), *Dictyostelium discoideum* (an eukaryote), and *Thermoplasma acidophilum* (an archaeon). We identified the nutrients that these organisms take up (Tables S2a–j in Text S1) from the prediction of nutrient transporters found in the database TransportDB [39] (accessed 11/03/2010).

#### Metabolites and pathways

We downloaded KEGG [33]–[35], and SEED's [28], [58] metabolite databases, and KEGG's pathway database. The SEED metabolite database allows us to link the metabolites in various reconstructions to their respective KEGG IDs. The KEGG metabolite and pathway databases allow us to assign the metabolic pathways to the individual metabolites in the reconstruction. The pathway database in KEGG is organized into 7 groups, one of which is metabolism. Metabolism is further divided into 11 major subgroups (for example, “Amino Acid Metabolism”). Each of these major subgroups is further divided into metabolic pathways (for example, “Arginine and Proline Metabolism”). There are 156 distinct metabolic pathways, but only 50 pathways are found in *all* seven species we used in this study.

We cross-checked the identifiers used in the reconstructions with the linked names in the SEED database to find mistakes, duplicates, and missing KEGG IDs. There are metabolites used in the reconstructions that we could not find in the KEGG database. For each one of these, we attempted to manually assign the KEGG ID of related metabolites, by using KEGG and SEED. There were 27 metabolites for which we attempted this manual assignment; for 5 of these, we could not unambiguously assign a KEGG ID (Tables S3a&b in Text S1). In addition, there are metabolites which do not have any KEGG pathways assigned. We used KEGG and SEED to find the most relevant pathway for these metabolites, and we manually assigned the KEGG ID of another metabolite in the same pathway, thereby assigning the same pathways by default. There were 43 metabolites for which we attempted this manual assignment. For 12 of these, we were unable to find an appropriate pathway classification (Tables S3c&d in Text S1).

#### Complex nutrients

Many of the 352 nutrients in our list may be considered complex in that they are composed of one or more simple nutrients. For example, adenosine consists of two simple nutrients: adenine and ribose. Complex nutrients are typically degraded into their simple components and then each component may or may not be catabolized separately. We denoted every nutrient in our list either as simple or complex. For the latter, we identified the simple nutrients that compose it (Tables S4a–f in Text S1).

#### Nutrient classes

We classify the nutrients by their structure and, to a lesser extent, function. We define 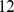 classes. Five of these classes—“Sugars”, “Amino acids” (including uncommon amino acids), “Purines”, “Pyrimidines”, and “Fatty acids”—are self-explanatory. We classify elements and sources of inorganic carbon as “Inorganic compounds.” The “Cofactors” class includes cofactors, vitamins—which are precursors of cofactors—and nutrients whose primary function is to chelate an inorganic element. Many nutrients that were deemed to be derivatives of amino acids, sugars or fatty acids were included in the relevant “derivative” categories. In addition, larger nutrients involved in the formation of the cell boundary, such as lipid A, were included in the “Cell boundary” nutrient class. We lumped together the remaining nutrients—which are small organic compounds—as “Organic compounds” (Tables S5a–l in Text S1).

### Flux Balance Analysis experiments

Stoichiometric methods have been widely used in metabolic engineering for over 20 years [59], the most used of which is Flux Balance Analysis (FBA) [26], [27], [60]. FBA aims at determining the fluxes 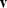 through each one of the metabolic reactions in an organism. Thus, FBA relies on the determination of a stoichiometric matrix 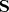 that represents all the reactions and metabolites in an *in silico* organism 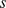, and a vector 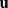 of uptake fluxes.

In the matrix 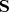, each row corresponds to a reaction, and each column to a metabolite. 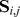 is the stoichiometric coefficient of metabolite 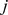 in reaction 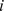. The vector of uptake fluxes 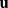 can have a non-zero entry for every transport reaction that moves a nutrient into the *in silico* organism.

As a simple example, consider a metabolic network with three reactions:
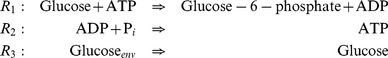
(7)where 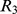 represents the transport reaction for uptaking Glucose from the environment. The stoichiometric matrix for metabolic network (7) is:
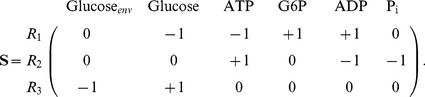
(8)Assuming a steady-state concentration of every metabolite and requiring mass-conservation we must impose that
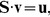
(9)where 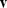 is the set of fluxes through each metabolic reaction. In order to solve Eq. (9), we need to provide the vector 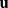. The default flux for each uptake reaction is 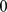, meaning that the nutrient is not in the medium or that it cannot be uptaken by the organism. We set 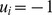 if nutrient 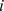 is present in the medium and could be taken up by the organism.

The system in Eq. (9) has many solutions. In our analysis, the biologically relevant metabolic state is the one that maximizes biomass production 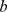 [59]. The problem of finding the fluxes through the reactions with a cost function that have to satisfy a number of constraints is a standard linear optimization problem that can be numerically solved using the subroutines provided in GLPK [61].

In order for us to model whether a nutrient can be a source of carbon and the biomass yield of the nutrient, we control the data in two ways. First, for some organisms the uptake of specific nutrients can lead to 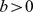 without being considered significant [8]. Secondly, ATP hydrolysis is integrated into the biomass of the *in silico* organisms, and is typically trained to better match empirical results based on growth rates. We are not exploring growth rates and we thus adjust the ATP hydrolysis in the biomass of the *in silico* organisms in order to better support the conclusions we reach. These controls are explained in detail in Text S1.

### Model selection

#### Model accuracy

To determine which of the logistic models we build is the best, we must balance accuracy with economy. We assess the model's sensitivity and specificity using two sets of measures. First, we calculate the true positive rate (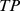) and the true negative rate (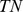), which indicate how many nutrients are successfully predicted to be in 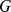, or 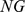, respectively. Secondly, we calculate the area under the ROC curve, 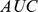, to quantify the accuracy of the model [62].

We assess whether the prediction for a nutrient is a true positive or a true negative as follows: For each nutrient 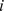, we first estimate 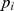. For every 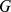 nutrient, if 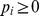 (which is equivalent to 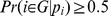), the nutrient is predicted to be a source of carbon, and we record a true positive. If, for a 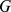 nutrient, 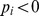, the nutrient is predicted to not be a source of carbon and we record a false negative. For every 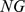 nutrient, if 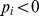, it is recorded as a true negative; if 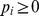, it is recorded as a false positive.

We calculate the 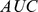 as follows. First, we rank all of the nutrients according to the probability that they are a source of carbon 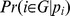. Then starting with the nutrient with the highest 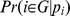, for every 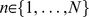 nutrients we count the fraction of nutrients that are sources of carbon 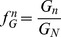 and the fraction of nutrients that are not sources of carbon 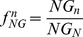 where 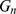 and 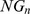 are the number of nutrients in 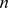 that can, and cannot be sources of carbon respectively, and 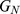 and 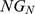 are the total number of nutrients that can and cannot be sources of carbon, respectively. Each (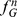,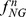) pair is a point in the 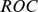 curve. We expect 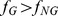 at the top of the list and 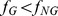 at the bottom of the list. The 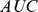 is the area under this curve. An 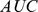 close to 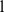 means that the pathways model succeeded in separating the nutrients that are sources of carbon from the nutrients that are not sources of carbon.

#### Model economy

The larger 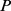 and 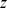 are (that is, the larger the number of parameters in Eq. (2), the more accurate the model is for the data at hand. However, with every additional parameter, the chances that we overfit the data increases. This issue becomes particularly worrisome if we consider pathways that are only partially conserved, for these pathways may not be represented in the test set of species we use to validate our model. To assess the economy of the model, we use two standard information criteria, the Akaike Information Criterion (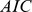) [63]

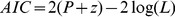
(10)and the Bayesian Information Criterion (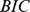) [64]

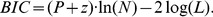
(11)


These information criteria balance the number of parameters, which in this case is the number of pathways and pairs of pathways 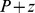, with the maximum likelihood of the model 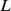, a measure of how effectively the model predicts the catabolic potential of a nutrient. When a new parameter is added to the model, 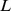 must increase sufficiently to balance the increase in 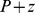. In addition, 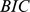 includes the number of data points (in our case nutrients 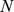 in the dataset), and is therefore more stringent if the dataset is small. By adding parameters, we can find the pathways and pairs of pathways at which the information criteria are at the global minimum.

### Complex media

We tested our model on a large number of complex media generated using the nutrients available for uptake in the *in silico* organisms. Because the number of possible combinations of these nutrients was too large for us to test each one computationally, we considered an ensemble of 1000 randomly generated complex media 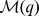. For each complex medium, every nutrient was made available for uptake with the probability 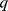, and was excluded with the probability 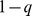. We generated ensembles for 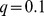, 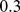, and 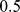 (for a total of 3000 random media).

Sugars present an unusual case because a microbe such as *E. coli* has been known to exhibit diauxic growth [65]–[67]. This means that microbes regulate sugar uptake so that, despite having various sugars present in the medium, they will only take up one sugar at a time. Our model does not take gene regulation into account, and therefore we manually limited the number of sugars presents in each complex medium to one, specifically glucose.

## Supporting Information

Text S1 **Supplementary information regarding data controls.** Supplementary figures showing: S1) the redundancy in pathway membership; S2) the model selection process. Tables describing: S1) the minimal media used for each species; S2) the prediction of our model for each nutrient separated by nutrient type; S3) the reassignment of KEGG IDs and pathways for compounds lacking that information; S4) the composition of complex nutrients; S5) the classification of nutrients in different classes; S6) the uptake of fatty acids and sugars by the different species; S7) the prediction of the logistic regression pathway model for each nutrient; S8) coefficients for the different terms in the logistic regression model.
(PDF)

## References

[1] Stephanopoulos G (2007) Challenges in engineering microbes for biofuels production. Science 315: 801–804. 17289987 10.1126/science.1139612

[2] Timmis KN, Steffan RJ, Unterman R (1994) Designing microorganisms for the treatment of toxic wastes. Annual Review of Microbiology 48: 525–557. 10.1146/annurev.mi.48.100194.002521 7826017

[3] Faulwetter JL, Gagnon V, Sundberg C, Chazarenc F, Burr MD, et al (2009) Microbial processes influencing performance of treatment wetlands: A review. Ecological Engineering 35: 987–1004.

[4] Keasling JD (2010) Manufacturing molecules through metabolic engineering. Science 330: 1355–1358. 21127247 10.1126/science.1193990

[5] Levine AJ, Puzio-Kuter AM (2010) The control of the metabolic switch in cancers by oncogenes and tumor suppressor genes. Science 330: 1340–1344. 21127244 10.1126/science.1193494

[6] Rabinowitz JD, White E (2010) Autophagy and metabolism. Science 330: 1344–1348. 21127245 10.1126/science.1193497 PMC3010857

[7] Handelsman J (2004) Metagenomics: application of genomics to uncultured microorganisms. Microbiol Mol Biol Rev 68: 669–685. 15590779 10.1128/MMBR.68.4.669-685.2004 PMC539003

[8] Feist AM, Henry CS, Reed JL, Krummenacker M, Joyce AR, et al (2007) A genome-scale metabolic reconstruction for Escherichia coli K-12 MG1655 that accounts for 1260 ORFs and thermodynamic information. Mol Syst Biol 3: 121. 17593909 10.1038/msb4100155 PMC1911197

[9] Oh YK, Palsson BØ, Park SM, Schilling CH, Mahadevan R (2007) Genome-scale reconstruction of metabolic network in Bacillus subtilis based on high-throughput phenotyping and gene essentiality data. J Biol Chem 282: 28791–28799. 17573341 10.1074/jbc.M703759200

[10] Bader FG (1978) Analysis of double-substrate limited growth. Biotechnol Bioeng 20: 183–202. 630068 10.1002/bit.260200203

[11] Egli T, Lendemann U, Snozzi M (1993) Kinetics of microbial growth with mixtures of carbon sources. Antonie Van Leeuwenhoek 63: 289–98. 8279825 10.1007/BF00871224

[12] Kovárová-Kovar K, Egli T (1998) Growth kinetics of suspended microbial cells: from singlesubstrate-controlled growth to mixed-substrate kinetics. Microbiol Mol Biol Rev 62: 646–666. 9729604 10.1128/mmbr.62.3.646-666.1998 PMC98929

[13] Toda K (2003) Theoretical and methodological studies of continuous microbial bioreactors. J Gen Appl Microbiol 49: 219–33. 14581991 10.2323/jgam.49.219

[14] Zinn M, Witholt B, Egli T (2004) Dual nutrient limited growth: models, experimental observations, and applications. J Biotechnol 113: 263–279. 15380660 10.1016/j.jbiotec.2004.03.030

[15] Cassman M, Arkin A, Katagiri F, Lauffenburger D, Doyle F, et al (2006) Barriers to progress in systems biology. Nature 438: 1079. 10.1038/4381079a 16371982

[16] Cassman M, Arkin A, Doyle F, Katagiri F, Lauffenburger D, et al.. (2007) Systems Biology. International Research and Development. Springer pp 35–40.

[17] Schuster P (2008) Modeling in biological chemistry. from biochemical kinetics to systems biology. Monatsh Chem 139: 427–446.

[18] Joshi A, Palsson BØ (1989) Metabolic dynamics in the human red cell. Part I–A comprehensive kinetic model. J Theor Biol 141: 515–528. 2630803 10.1016/s0022-5193(89)80233-4

[19] Adadi R, Volkmer B, Milo R, Heinemann M, Shlomi T (2012) Prediction of microbial growth rate versus biomass yield by a metabolic network with kinetic parameters. PLoS Comp Biol 8: e1002575. 10.1371/journal.pcbi.1002575 PMC3390398 22792053

[20] Thiele I, Vo TD, Price ND, Palsson BØ (2005) Expanded metabolic reconstruction of Helicobacter pylori (iIT341 GSM/GPR): an in silico genome-scale characterization of single- and double-deletion mutants. J Bacteriol 187: 5818–5830. 16077130 10.1128/JB.187.16.5818-5830.2005 PMC1196094

[21] Mo ML, Palsson BØ, Herrgård MJ (2009) Connecting extracellular metabolomic measurements to intracellular flux states in yeast. BMC Syst Biol 3: 37. 19321003 10.1186/1752-0509-3-37 PMC2679711

[22] Becker SA, Palsson BØ (2005) Genome-scale reconstruction of the metabolic network in Staphylo-coccus aureus N315: an initial draft to the two-dimensional annotation. BMC Microbiol 5: 8. 15752426 10.1186/1471-2180-5-8 PMC1079855

[23] Jamshidi N, Palsson BØ (2007) Investigating the metabolic capabilities of Mycobacterium tuber-culosis H37Rv using the in silico strain iNJ661 and proposing alternative drug targets. BMC Syst Biol 1: 26. 17555602 10.1186/1752-0509-1-26 PMC1925256

[24] Feist AM, Scholten JCM, Palsson BØ, Brockman FJ, Ideker T (2006) Modeling methanogenesis with a genome-scale metabolic reconstruction of Methanosarcina barkeri. Mol Syst Biol 2: 2006.0004. 10.1038/msb4100046 PMC1681478 16738551

[25] Varma A, Palsson BØ (1994) Stoichiometric flux balance models quantitatively predict growth and metabolic by-product secretion in wild-type Escherichia coli w3110. Appl Environ Microbiol 60: 3724–3731. 7986045 10.1128/aem.60.10.3724-3731.1994 PMC201879

[26] Edwards JS, Ibarra RU, Palsson BØ (2001) In silico predictions of Escherichia coli metabolic capabilities are consistent with experimental data. Nat Biotechnol 19: 125–130. 11175725 10.1038/84379

[27] Orth JD, Thiele I, Palsson BØ (2010) What is flux balance analysis? Nat Biotechnol 28: 245–248. 20212490 10.1038/nbt.1614 PMC3108565

[28] Overbeek R, Begley T, Butler RM, Choudhuri JV, Chuang HY, et al (2005) The subsystems approach to genome annotation and its use in the project to annotate 1000 genomes. Nucleic Acids Res 33: 5691–5702. 16214803 10.1093/nar/gki866 PMC1251668

[29] Reed JL, Vo TD, Schilling CH, Palsson BØ (2003) An expanded genome-scale model of Escherichia coli K-12 (iJR904 GSM/GPR). Genome Biol 4: R54. 12952533 10.1186/gb-2003-4-9-r54 PMC193654

[30] Monod J (1966) On the mechanism of molecular interactions in the control of cellular metabolism. Endocrinology 78: 412–425. 5324063 10.1210/endo-78-2-412

[31] Dong H, Nilsson L, Kurland CG (1996) Co-variation of tRNA abundance and codon usage in Escherichia coli at different growth rates. J Mol Biol 260: 649–663. 8709146 10.1006/jmbi.1996.0428

[32] Beg QK, Vazquez A, Ernst J, de Menezes MA, Bar-Joseph Z, et al (2007) Intracellular crowding defines the mode and sequence of substrate uptake by escherichia coli and constrains its metabolic activity. Proc Natl Acad Sci U S A 104: 12663–12668. 17652176 10.1073/pnas.0609845104 PMC1937523

[33] Kanehisa M, Goto S (2000) KEGG: Kyoto encyclopedia of genes and genomes. Nucleic Acids Res 28: 27–30. 10592173 10.1093/nar/28.1.27 PMC102409

[34] Kanehisa M, Goto S, Hattori M, Aoki-Kinoshita KF, Itoh M, et al (2006) From genomics to chemical genomics: New developments in KEGG. Nucleic Acids Res 34: D354–D357. 16381885 10.1093/nar/gkj102 PMC1347464

[35] Kanehisa M, Goto S, Furumichi M, Tanabe M, Hirakawa M (2010) KEGG for representation and analysis of molecular networks involving diseases and drugs. Nucleic Acids Res 38: D355–D360. 19880382 10.1093/nar/gkp896 PMC2808910

[36] Higgins CF (2001) ABC transporters: physiology, structure and mechanism–an overview. Res Microbiol 152: 205–210. 11421269 10.1016/s0923-2508(01)01193-7

[37] Moussatova A, Kandt C, O'Mara ML, Tieleman DP (2008) ATP-binding cassette transporters in Escherichia coli. Biochim Biophys Acta 1778: 1757–1771. 18634750 10.1016/j.bbamem.2008.06.009

[38] Ren Q, Paulsen IT (2007) Large-scale comparative genomic analyses of cytoplasmic membrane transport systems in prokaryotes. J Mol Microbiol Biotechnol 12: 165–179. 17587866 10.1159/000099639

[39] Ren Q, Chen K, Paulsen IT (2007) TransportDB: a comprehensive database resource for cytoplasmic membrane transport systems and outer membrane channels. Nucleic Acids Res 35: D274–D279. 17135193 10.1093/nar/gkl925 PMC1747178

[40] Kamp F, Hamilton JA (2006) How fatty acids of different chain length enter and leave cells by free diffusion. Prostaglandins Leukot Essent Fatty Acids 75: 149–159. 16829065 10.1016/j.plefa.2006.05.003

[41] Siebold C, Fl¨ukiger K, Beutler R, Erni B (2001) Carbohydrate transporters of the bacterial phosphoenolpyruvate: sugar phosphotransferase system (PTS). FEBS Lett 504: 104–111. 11532441 10.1016/s0014-5793(01)02705-3

[42] Leandro MJ, Fonseca C, Goņcalves P (2009) Hexose and pentose transport in ascomycetous yeasts: an overview. FEMS Yeast Res 9: 511–525. 19459982 10.1111/j.1567-1364.2009.00509.x

[43] Barker CJ, Illies C, Gaboardi GC, Berggren PO (2009) Inositol pyrophosphates: structure, enzymology and function. Cell Mol Life Sci 66: 3851–3871. 19714294 10.1007/s00018-009-0115-2 PMC11115731

[44] Reynolds TB (2009) Strategies for acquiring the phospholipid metabolite inositol in pathogenic bacteria, fungi and protozoa: making it and taking it. Microbiology 155: 1386–1396. 19383710 10.1099/mic.0.025718-0 PMC2889408

[45] Black PN, DiRusso CC (1994) Molecular and biochemical analyses of fatty acid transport, metabolism, and gene regulation in Escherichia coli. Biochim Biophys Acta 1210: 123–145. 8280762 10.1016/0005-2760(94)90113-9

[46] Gelman A, Hill J (2006) Data Analysis Using Regression and Multilevel/Hierarchical Models. Cambridge University Press. pp 79–108.

[47] R Development Core Team (2005) R: A Language and Environment for Statistical Computing. R Foundation for Statistical Computing. Viena (Austria).

[48] Wong WW, Tran LM, Liao JC (2009) A hidden square-root boundary between growth rate and biomass yield. Biotechnol Bioeng 102: 73–80. 18683253 10.1002/bit.22046

[49] Saier MH, Yen MR, Noto K, Tamang DG, Elkan C (2009) The transporter classification database: recent advances. Nucleic Acids Res 37: D274–D278. 19022853 10.1093/nar/gkn862 PMC2686586

[50] Yen MR, Choi J, Saier MH (2009) Bioinformatic analyses of transmembrane transport: novel software for deducing protein phylogeny, topology, and evolution. J Mol Microbiol Biotechnol 17: 163–176. 19776645 10.1159/000239667 PMC2814153

[51] Guimerà R, Amaral LAN (2005) Cartography of complex networks: modules and universal roles. J Stat Mech (P02001) nihpa35573. 10.1088/1742-5468/2005/02/P02001 PMC2151742 18159217

[52] Guimerà R, Amaral LAN (2005) Functional cartography of complex metabolic networks. Nature 433: 895–900. 15729348 10.1038/nature03288 PMC2175124

[53] Sales-Pardo M, Guimerà R, Moreira AA, Amaral LAN (2007) Extracting the hierarchical organization of complex systems. Proc Natl Acad Sci U S A 104: 15224–15229. 17881571 10.1073/pnas.0703740104 PMC2000510

[54] Sambrook J, Russell D, editors (2001) Molecular Cloning: A Laboratory Manual. Cold Spring Harbor Lab. Press Ap A2.1.

[55] Guthrie C, Fink GR, editors (1991) Guide to Yeast Genetics and Molecular Biology. Academic. p 50.

[56] Demain AL (1958) Minimal media for quantitative studies with Bacillus subtilis. J Bacteriol 75: 517–522. 13538918 10.1128/jb.75.5.517-522.1958 PMC290103

[57] Reynolds DJ, Penn CW (1994) Characteristics of Helicobacter pylori growth in a defined medium and determination of its amino acid requirements. Microbiology 140: 2649–2656. 8000535 10.1099/00221287-140-10-2649

[58] Henry CS, DeJongh M, Best AA, Frybarger PM, Linsay B, et al (2010) High-throughput generation, optimization and analysis of genome-scale metabolic models. Nat Biotechnol 28: 977–982. 20802497 10.1038/nbt.1672

[59] Palsson BØ (2006) Systems biology: Properties of reconstructed networks. Cambridge, MA, USA: Cambridge University Press. pp 89–178.

[60] Varma A, Boesch BW, Palsson BØ (1993) Stoichiometric interpretation of Escherichia coli glucose catabolism under various oxygenation rates. Appl Environ Microbiol 59: 2465–2473. 8368835 10.1128/aem.59.8.2465-2473.1993 PMC182307

[61] Makhorin A (2009). Linear Programming Kit, Version 4.40. GNU.

[62] Hanley J, McNeil B (1982) The meaning and use of the area under a receiver operating characteristic (ROC) curve. Radiology 143: 29–36. 7063747 10.1148/radiology.143.1.7063747

[63] Akaike H (1974) A new look at the statistical model identification. IEEE Transactions on Automatic Control 19: 716–723.

[64] Schwarz G (1978) Estimating the dimension of a model. Ann Stat 6: 461–464.

[65] Monod J (1942) The growth of bacterial cultures. Annu Rev Microbiol 3: 371–394.

[66] Kornberg H, Watts PD, Brown K (1980) Mechanisms of ‘inducer exclusion’ by glucose. FEBS Lett 117 Suppl: K28–K36. 6252047 10.1016/0014-5793(80)80567-9

[67] Postma PW, Lengeler JW, Jacobson GR (1993) Phosphoenolpyruvate:carbohydrate phosphotransferase systems of bacteria. Microbiol Rev 57: 543–594. 8246840 10.1128/mr.57.3.543-594.1993 PMC372926

[68] Liu Y, Whitman WB (2008) Metabolic, phylogenetic, and ecological diversity of the methanogenic archaea. Ann N Y Acad Sci 1125: 171–189. 18378594 10.1196/annals.1419.019

