# Supplementary material for: Phenomenological Model for Predicting the Catabolic Potential of an Arbitrary Nutrient
Source: PLoS Comput Biol. 2012 Nov 1;8(11):e1002762. doi: 10.1371/journal.pcbi.1002762 (PMC3486842; doi:10.1371/journal.pcbi.1002762)
Supplement: Text S1 — Supplementary information regarding data controls. Supplementary figures showing: S1) the redundancy in pathway membership; S2) the model selection process. Tables describing: S1) the minimal media used for each species; S2) the prediction of our model for each nutrient separated by nutrient type; S3) the reassignment of KEGG IDs and pathways for compounds lacking that information; S4) the composition of complex nutrients; S5) the classification of nutrients in different classes; S6) the uptake of fatty acids and sugars by the different species; S7) the prediction of the logistic regression pathway model for each nutrient; S8) coefficients for the different terms in the logistic regression model. (PDF) [file pcbi.1002762.s001.pdf]

# Text S1

S. M. D. Seaver[1,2], M. Sales-Pardo[3,1], R. Guimer[4,3,1], L. A. N. Amaral[1,2,4,*]
**1 Department of Chemical and Biological Engineering, Northwestern University, Evanston, IL 60208, USA**
**2 Interdisclipinary Biological Sciences, Northwestern University, Evanston, IL 60208, USA**
**3 Department d'Enginyeria Química, Universitat Rovira i Virgill, 43007 Tarragona, Spain**
**4 Institució Catalana de Recerca i Estudis Avançats (ICREA), 08010 Barcelona, Catalonia**
**5 Howard Hughes Medical Institute, Northwestern University, Evanston, IL 60208, USA**

## Supplementary Text

## Data controls

In what follows, we describe how we modified the ATP hydrolysis in the *in silico* organisms so that biomass production reflected nutrient catabolysis, and how we identified true biomass production from the results of FBA experiments.

**ATP maintenance.** Some of the *in silico* organisms were constructed to directly predict the *in vivo* growth rate, which is typically lower than the predicted biomass yield. The growth rate of an organism is related to the biomass yield, but it is a kinetic property that is affected by a variety of factors such as nutrient uptake, regulation of protein expression, and temperature. The biomass yield is dimensionless and reflects the efficiency with which the organism uses the nutrients available [1]. The authors of the *in silico* organisms model the growth rate by forcing the *in silico* organism to hydrolyze a fixed amount of extra ATP, known as ATP maintenance. The ATP maintenance is typically calculated by fitting growth rate data of the organism on a single medium [2]. As we are only modeling biomass yield, we remove ATP maintenance from the *in silico* organisms as follows.

There are two types of maintenance, growth-associated maintenance (GAM), and non-growth associated maintenance (NGAM). For NGAM, the hydrolysis of ATP is added as a separate reaction, and this merely changes the lower bound of nutrient uptake that is required for biomass production to begin (see section on $b_{min}$ below). We turn off this reaction if it is present by constraining its flux to zero.

For GAM, the hydrolysis of ATP is incorporated into the biomass function, and this affects the overall growth rate of the *in silico* organism. However, the ATP hydrolysis in the biomass function accounts for both GAM and ATP needed to polymerize protein and DNA. The energetic costs of the polymerization of biomass components is important for biomass yield, and we therefore cannot remove the ATP hydrolysis from the biomass function entirely.

To solve this problem, we calculate the ATP needed for the polymerization of biomass components using the experimentally determined values published for *E. coli* [2, 3]. The published biomass function for each *in silico* organism contains the stoichiometry of the protein, DNA, and RNA, and it is straightforward to calculate the stoichimetric coefficient for ATP hydrolysis needed for polymerization costs alone. We replace the published coefficient for ATP hydrolysis with the calculated value. For *E. coli* and *S. cerevisiae*, this value of ATP needed for polymerization costs was already available [2, 4, 5].

**Source of carbon.** Consider a species $s$ growing on nutrient $i$. By using FBA, we find the optimal biomass produced $b_i^s$. Often $b_i^s > 0$, but to decide whether it can be considered true biomass production, we have to take into account the following issues:

- *H. pylori* and *M. tuberculosis* present the unusual case of having nutrients in the minimal medium with which they can already produce biomass in the absence of any additional nutrients. This means that we will observe biomass production for any nutrient we test, even if the nutrient cannot be catabolized. We counter this by introducing a minimal biomass production, $b^s_{min}$. Any biomass production in excess of $b^s_{min}$ is then attributable to the nutrient we are testing.

- In the latest reconstruction of *E. coli*, there are several nutrients for which Feist *et al.* considered the resulting biomass to be too small [2]. Additionally, when considering an organism that has $b^s_{min} > 0$, some nutrients such as pyrimidines will produce biomass above $b^s_{min}$ even though they are not catabolized. The reason for this is that these nutrients are directly used in the biomass, i.e. these nutrients are not catabolized, and thereby save the organism carbon and energy, resulting in a larger biomass production. For these reasons, we estimated the minimal biomass production threshold $b_{cat}$ beyond which biomass production is attributable to a nutrient being catabolized. We set $b_{cat} = 0.008$, and assume that nutrients for which $b^s_i - b^s_{min} < b_{cat}$ are not catabolized.

In building our model, our first concern is to determine whether a nutrient can be a source of carbon. Therefore, we reduce the biomass production $b^s_i$ to a binary observation $\alpha^s_i$ such that:

$$\alpha^s_i = \begin{cases} NG & \text{if } b^s_i \leq b^s_{min} + b_{cat} \\ G & \text{if } b^s_i > b^s_{min} + b_{cat} \end{cases} \tag{1}$$

In the reconstructions of *E. coli* and *H. pylori*, there are five nutrients available that were added as sinks for metabolites that had been observed to accumulate *in silico* [2, 6]. We do not consider these nutrients in our analysis.

The biomass reaction for *S. aureus* was derived from the same source as *B. subtilis*, but it was designed so that its demand in the number of carbons is approximately 100-fold higher than that of *B. subtilis.* If we reduce the observation to a binary variable, this should not matter, even if the resulting biomass produced is 100-fold smaller. However, for some nutrients, FBA fails to reach a solution because the maximum nutrient uptake value of $-1$ is too small. We counter this by using a maximum nutrient uptake value of $-100$ in *S. aureus.*

## Supplementary Figures

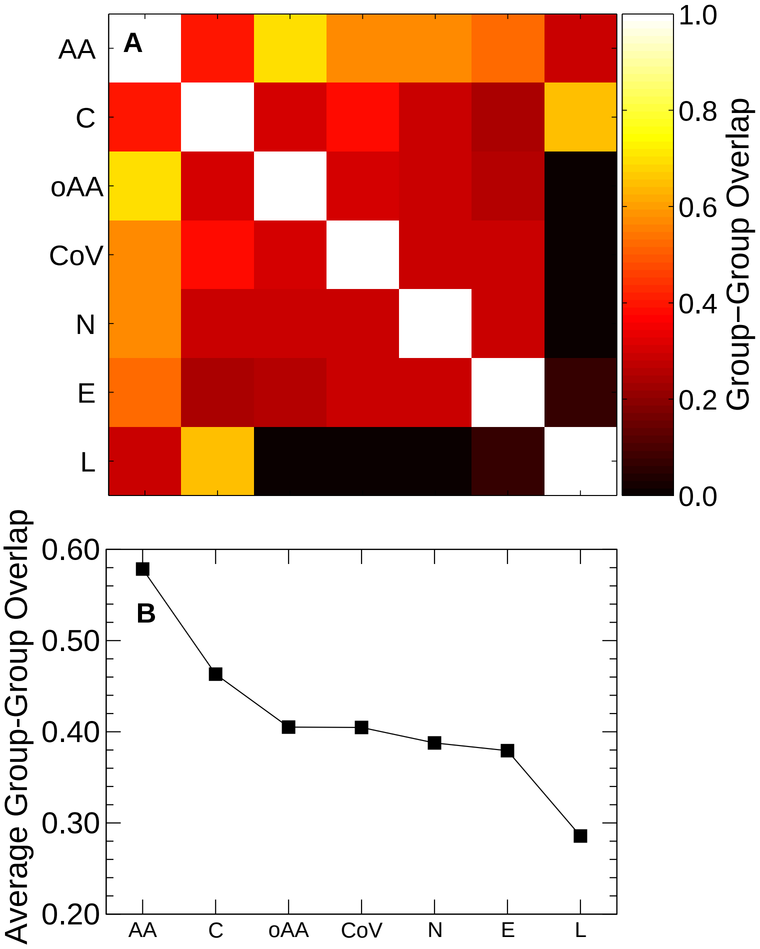


**Figure S1. Redundancy of nutrient-pathway membership. A** The overlap for pairs of groups. Many nutrients are found in more than one KEGG pathway, and also in more than one group of pathways. This redundancy is depicted here as group-group overlap. For each pair of groups, we count the number of nutrients that are members of pathways found in both groups, and normalize with the number of nutrients in the smaller group. We only perform this analysis on nutrients which are not classified as $G$ or $NG$ based on chemical structure and function (see main text). **B** Average overlap for individual groups. We find that Amino acid metabolism (AA) has the highest average overlap, sharing many nutrients with other groups. This finding is consistent with the notion that AA is central to metabolism. Key: AA=Amino acid metabolism; C=Carbohydrate metabolism; oAA=Metabolism of other amino acids; CoV=Metabolism of cofactors and vitamins; N=Nucleotide metabolism; E=Energy metabolism; L=Lipid metabolism.

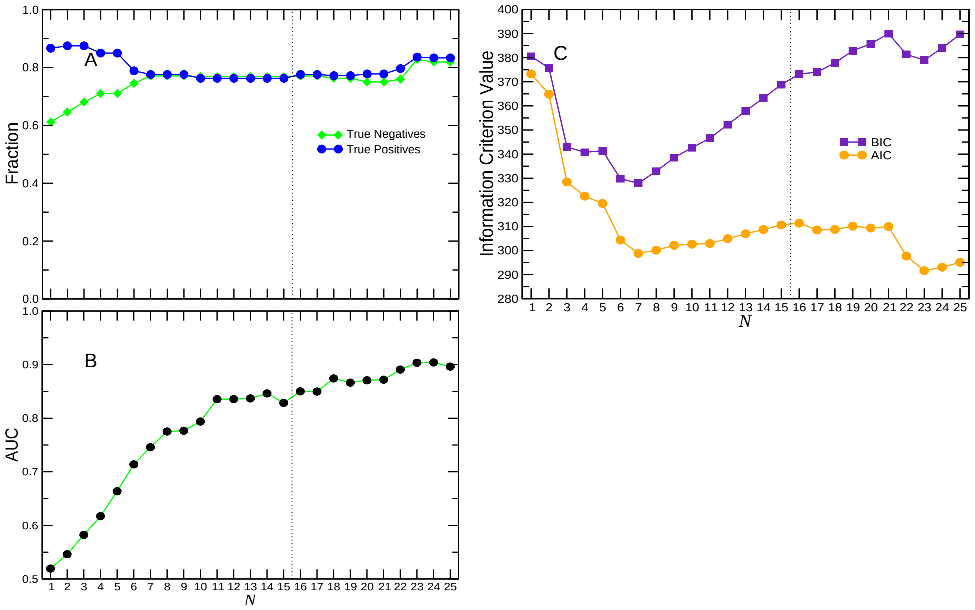


**Figure S2. Model selection.** We generate logistic models according to the pathways listed in supplemental table S6. We focus first on the $G$ nutrients (15 pathways) and only then add the 11 pathways comprising mostly $NG$ nutrients. Note how for $N > 7$ the values of the information criteria start increasing indicating that the model is overfitting the data.

## Supplementary Tables

### Supplemental Table S1: Minimal media for *in silico* organisms.

The presence of a symbol indicates that a species uptakes the nutrient in question. The symbol type indicates whether a nutrient's uptake is constrained: (
*checkmark*) a nutrient's uptake is unconstrained, ($n$) a nutrient's uptake is constrained by $n$. Such nutrients contain organic carbon and the value for $n$ is taken from the biomass function.

In the case of cytosine in *S. aureus*, $n$ is the sum of the CMP and dCMP requirements in the biomass. *M. barkeri* respires anaerobically, and thus does not have oxygen in its minimal media. In addition, *M. barkeri*'s source of sulfur is $SO_3^{-2}$ and its terminal electron acceptor is $H_2S$.

Key: *Bs*=*B. subtilis*, *Ec*=*E. coli*, *Mb*=*M. barkeri*, *Sc*=*S. cerevisiae*, *Sa*=*S. aureus*, *Mt*=*M. tuberculosis*, *Hp*=*H. pylori*.

**Table S1. Minimal media for *in silico* organisms.**

| Name | *Bs* | *Ec* | *Mb* | *Sc* | *Sa* | *Mt* | *Hp* |
|---|---|---|---|---|---|---|---|
| $H_2O$ | ✓ | ✓ | ✓ | ✓ | ✓ | ✓ | ✓ |
| $O_2$ | ✓ | ✓ | | ✓ | ✓ | ✓ | ✓ |
| $CO_2$ | ✓ | | | | | | |
| $H^+$ | ✓ | ✓ | ✓ | ✓ | ✓ | ✓ | ✓ |
| $NH_4{}^+$ | ✓ | ✓ | ✓ | ✓ | ✓ | ✓ | ✓ |
| $SO_4{}^{-2}$ | ✓ | ✓ | | ✓ | ✓ | ✓ | ✓ |
| $SO_3{}^{-2}$ | | | ✓ | | | | |
| $H_2S$ | | | ✓ | | | | |
| $HPO_4{}^{-2}$ | ✓ | ✓ | ✓ | ✓ | ✓ | ✓ | ✓ |
| $MoO_4{}^{-2}$ | | ✓ | | | | | |
| $K^+$ | ✓ | ✓ | | | | ✓ | |
| $Na^+$ | ✓ | ✓ | ✓ | ✓ | | ✓ | |
| $Ca^{+2}$ | ✓ | ✓ | | | | ✓ | |
| $Co^{+2}$ | | ✓ | ✓ | | | | |
| $Cu^{+2}$ | | ✓ | | | | ✓ | |
| $Fe^{+2}$ | | ✓ | | ✓ | ✓ | | ✓ |
| $Mg^{+2}$ | ✓ | ✓ | | | | | |
| $Ni^{+2}$ | | | ✓ | | | | |
| $Zn^{+2}$ | | ✓ | | | | | |
| $Fe^{+3}$ | ✓ | ✓ | | | | ✓ | ✓ |
| $Mn^{+3}$ | | ✓ | | | | | |
| $Cl^-$ | | ✓ | | | | ✓ | |
| Cob(I)alamin | | ✓ | | | | | |
| Cytosine | | | | | −2.73 | | |
| Glycine | | | | | −3.51 | | |
| L-Alanine | | | | | | | −0.488 |
| L-Valine | | | | | | | −0.402 |
| L-Leucine | | | | | | | −0.428 |
| L-Isoleucine | | | | | | | −0.276 |
| L-Arginine | | | | | | | −0.281 |
| L-Methionine | | | | | | | −0.146 |
| L-Histidine | | | | | | | −0.090 |
| Pimelate | | | | | | | $-6e^{-6}$ |
| Thiamin | | | | | −0.01 | | $-6e^{-6}$ |
| Niacin | | | −0.0027 | | −0.01 | | |
| Glycerol | | | | | | −0.0254 | |
| 4-Aminobenzoate | | | −0.0237 | | | | |

**Supplemental Table S2: Nutrient predictions.**

The presence of a symbol indicates that a species uptakes the nutrient in question. The symbol type indicates whether the uptaken nutrient can be a source of carbon in the species in question: (✓) source of carbon, (x) not a source of carbon.

**Table S2(a). Predictions for "Purines" & "Pyrimidines."**

| Name | *R. palustris* | *L. monocytogenes* | *D. dictysoideum* | *T. acidophilum* |
|---|---|---|---|---|
| Adenine | | | x | x |
| Cytosine | | x | | x |
| Uracil | | x | | |
| Xanthine | | x | x | |

**Table S2(b). Predictions for "Fatty acids."**

| Name | *R. palustris* | *L. monocytogenes* | *D. dictysoideum* | *T. acidophilum* |
|---|---|---|---|---|
| Fatty acids | x | x | ✓ | x |

**Table S2(c). Predictions for "Cell boundary compounds."**

| Name | *R. palustris* | *L. monocytogenes* | *D. dictysoideum* | *T. acidophilum* |
|---|---|---|---|---|
| UDP-N-acetylglucosamine | | | x | |
| UDP-N-acetylgalactosamine | | | x | |
| Lipoprotein | x | | | |
| Phospholipid | | | x | |
| Teichoic acid | | x | | |

**Table S2(d). Predictions for "Cofactors."**

| Name | *R. palustris* | *L. monocytogenes* | *D. dictysoideum* | *T. acidophilum* |
|---|---|---|---|---|
| Acetly-CoA | | | x | |
| Biotin | | | x | |
| Ferrichome | | x | | |
| Glutathione | | | x | |
| Heme | x | | | |
| Hemin | x | | | |
| Sialic acid | | | x | |
| Pantothenate | | | | x |

**Table S2(e). Predictions for "Sugar derivatives."**

| Name | *R. palustris* | *L. monocytogenes* | *D. dictysoideum* | *T. acidophilum* |
|---|---|---|---|---|
| D-Glucarate | | ✓ | | |
| GDP-Fucose | | | ✓ | |
| UDP-Galactose | | | ✓ | |
| Galactitol | | ✓ | | |
| Glucarate | | | | ✓ |
| Sorbitol | | ✓ | | |
| Glutamine | | ✓ | | |
| Galacturonate | ✓ | | | |
| Mannitol | | ✓ | | |
| Xylitol | | ✓ | | |
| Glucose-6-phosphate | | | | ✓ |

**Table S2(f). Predictions for "Sugars."**

| Name | *R. palustris* | *L. monocytogenes* | *D. dictysoideum* | *T. acidophilum* |
|---|---|---|---|---|
| 1,3-$\beta$-D-Glucan | ✓ | ✓ | | |
| Cellobiose | | ✓ | | |
| Fructose | | ✓ | | |
| Galactose | | | ✓ | |
| Glucose | ✓ | ✓ | ✓ | ✓ |
| Maltose | | ✓ | | |
| Mannose | ✓ | ✓ | | |
| Sucrose | | | ✓ | |
| Trehalose | ✓ | ✓ | | ✓ |

**Table S2(g). Predictions for "Inorganic compounds."**

| Name | *R. palustris* | *L. monocytogenes* | *D. dictysoideum* | *T. acidophilum* |
|---|---|---|---|---|
| $Ca^{+2}$ | x | x | x | |
| $SO_3{}^{-2}$ | x | x | x | |
| $Al^{+3}$ | | | x | |
| $NH_4{}^{+}$ | x | x | x | x |
| $H_3O_3As$ | x | x | x | |
| $Cd_2{}^{+}$ | | x | | |
| $Cl^{-}$ | x | x | x | x |
| $H_2O_4Cr$ | x | | | |
| $Co^{+2}$ | | x | | |
| $Cu^{+2}$ | x | x | x | x |
| $Fe^{+3}$ | x | x | | x |
| Pb | x | | x | |
| $Mg^{+2}$ | x | x | x | |
| $Mn^{+3}$ | x | x | x | x |
| $Hg_2{}^{+}$ | | x | | |
| $MoO_4{}^{-2}$ | x | x | | x |
| $Ni^{+2}$ | x | | | x |
| $NO_3{}^{-}$ | x | | | x |
| $HPO_4{}^{-2}$ | x | x | x | x |
| $K^{+}$ | x | x | x | x |
| $H^{+}$ | x | x | x | x |
| $Na^{+}$ | x | x | x | x |
| $H_2O$ | x | | | |
| $Zn^{+2}$ | | x | x | |

**Table S2(h). Predictions for "Organic compounds."**

| Name | *R. palustris* | *L. monocytogenes* | *D. dictysoideum* | *T. acidophilum* |
|---|---|---|---|---|
| $\alpha$-Ketoglutarate | | | ✓ | |
| 3-Phenylpropionate | x | | | |
| Toluene | x | | x | |
| Succinate | ✓ | | | |
| (S)-Lactate | ✓ | | | |
| Dracylic acid | x | | | |
| Glycine betaine | ✓ | ✓ | | ✓ |
| L-Carnitine | x | x | x | |
| Bilineurine | ✓ | ✓ | ✓ | |
| Folate | | | x | |
| Formate | x | x | x | x |
| Fumarate | | | ✓ | |
| Glycerol | | ✓ | ✓ | |
| Glycerol 3-phosphate | ✓ | | | |
| L-Malate | | | ✓ | |
| Malonate | x | | | |
| Muconate | x | | | |
| Oxalic acid | ✓ | ✓ | ✓ | ✓ |
| Oxaloacetate | | | ✓ | |
| Phosphoenolpyruvate | | | x | |
| 1,4-Butanediamine | ✓ | ✓ | | |
| Shikimate | x | | | |
| Acetamide | ✓ | | | |
| Spermidine | x | x | | |
| Succinate | | | ✓ | |
| Taurine | x | | | |
| Citrate | ✓ | | | |
| Urea | x | | x | |

**Table S2(i). Predictions for "Amino acids."**

| Name | *R. palustris* | *L. monocytogenes* | *D. dictysoideum* | *T. acidophilum* |
|---|---|---|---|---|
| Glycine | ✓ | ✓ | ✓ | ✓ |
| L-Alanine | ✓ | ✓ | ✓ | ✓ |
| D-Alanine | x | x | x | x |
| L-Serine | ✓ | ✓ | ✓ | ✓ |
| D-Serine | ✓ | ✓ | ✓ | ✓ |
| L-Threonine | ✓ | ✓ | ✓ | ✓ |
| L-Glutamate | ✓ | ✓ | ✓ | ✓ |
| D-Glutamate | x | x | x | x |
| L-Glutamine | ✓ | ✓ | ✓ | ✓ |
| L-Aspartate | ✓ | ✓ | ✓ | ✓ |
| L-Asparagine | ✓ | ✓ | ✓ | ✓ |
| L-Proline | ✓ | ✓ | ✓ | ✓ |
| L-Arginine | ✓ | ✓ | ✓ | ✓ |
| L-Cysteine | ✓ | ✓ | ✓ | ✓ |
| D-Cysteine | x | x | x | x |
| L-Methionine | x | x | x | x |
| D-Methionine | x | x | x | x |
| L-Leucine | x | x | x | x |
| L-Isoleucine | x | x | x | x |
| L-Valine | x | x | x | x |
| L-Lysine | x | x | x | x |
| L-Histidine | x | x | x | x |
| L-Phenylalanine | x | x | x | x |
| L-Tyrosine | x | x | x | x |
| L-Tryptophan | ✓ | ✓ | ✓ | ✓ |

**Supplemental Table S3: Reassignment of KEGG IDs and Pathways for nutrients in the *in silico* organisms.**

**Table S3(a). Simple compounds with reassigned KEGG IDs.**

| Name | Reassigned KEGG ID |
|---|---|
| 2-Methyl-1-butanol | C00233 |
| 2-Methylbutyl acetate | C00233 |
| 2-Methylpropanal | C00233 |
| 3-Aminobutanoate | C00334 |
| Butanesulfonate | C01412 |
| Butanoate | C00246 |
| Ethanesulfonate | C00084 |
| Hexacosanoate | C08320 |
| Hexadecenoate | C08362 |
| Hexanoate | C01585 |
| Isobutyl acetate | C00233 |
| L-Methionine R-Oxide | C02989 |
| L-Methionine-R-Sulfoxide | C00073 |
| L-Methionine-S-Sulfoxide | C00073 |
| Maltoheptaose | C01935 |
| Maltopentaose | C01935 |
| Monomethylamine | C00543 |
| Octadecanoate | C01530 |
| Octadecenoate | C00712 |
| Octadecynoate | C01595 |
| Tetradecenoate | C08322 |
| $\gamma$-Butyro-betaine | C00487 |

**Table S3(b). Simple compounds lacking a KEGG ID.**

| Name |
|---|
| MOPS |
| Hexanesulfonate |
| Arseno-betaine |
| $\alpha$-Methyl-D-glucoside |
| $\beta$-Methylglucoside |

**Table S3(c). Simple compounds with reassigned KEGG Pathways.**

| Name | Reassigned KEGG Pathway |
|---|---|
| (R)-Propane-1,2-diol | C00583 |
| 2,3-Diaminopropionate | C00163 |
| 2-Methylbutanal | C00671 |
| 5-Dehydro-D-gluconate | C00618 |
| Acetamide | C00033 |
| Butyro-betaine | C00487 |
| Crotono-betaine | C00487 |
| D-Allose | C00085 |
| D-Arabinose | C01112, C00309 |
| D-Gulitol | C00794 |
| D-Mannosamine | C00645 |
| D-Methionine | C00073 |
| D-O-Phosphoserine | C00740 |
| D-Psicose | C00085 |
| Glycerol 2-phosphate | C00116 |
| Glycerophosphoglycerol | C00093 |
| Glycogen | C01935 |
| Hexanoate | C05270 |
| L-Idonate | C00618 |
| Maltohexaose | C01935 |
| Maltotetraose | C01935 |
| Maltotriose | C01935 |
| Mercaptoacetate | C00155 |
| Methanesulfonate | C00409 |
| Methyl $\beta$-D-galactopyranoside | C01019 |
| Palatinose | C00089 |
| Phosphonotyrosine | C00082 |
| S-Methyl-L-Methionine | C00019 |
| Saligenin | C01451 |
| Tetradecenoate | C06424 |
| $\beta$-D-Galactose | C00124 |

**Table S3(d). Simple compounds lacking a KEGG Pathway.**

| Name |
| --- |
| Methyl sulfide |
| 2-Deoxy-D-glucose 6-phosphate |
| Isopentyl alcohol |
| Isovaleraldehyde |
| Gentiobiose |
| L-Djenkolic acid |
| Proline betaine |
| Dimethyl sulfoxide |
| Isoamyl acetate |
| Phenethyl acetate |
| Isobutyl alcohol |
| Acetyl ester |

**Supplemental Table S4: Composition of complex nutrients.**

The simple nutrients that compose each complex nutrients are listed here for all the species which take up the complex nutrient. The absence of a symbol indicates that the species does not take up the nutrient in question. x) The species takes up the nutrient but does not catabolize it; ○) The species takes up the nutrient but does not catabolize all of the simple nutrients that compose it; ✓) The species takes up the nutrient and catabolizes all of its components.

**Table S4(a). Composition of complex nutrients in the "Amino acids" class**

| Family | Name | *Ec* |
|---|---|---|
| Pyruvate, Indole | Tryptophan | |

**Table S4(b). Composition of complex nutrients in the "Fatty acid derivatives" class**

| Family | Name | *Ec* | *Sc* |
|---|---|---|---|
| Glycerol, myo-Inositol | 1(Glycerol-3-Phospho)-myo-inositol | ○ | x |
| Glycerol, Aminoethanol | Glycerophosphoethanolamine | ✓ | |
| Bilineurine, Glycerol | Glycerophosphocholine | ○ | x |
| L-Serine, Glycerol | Glycerophosphoserine | ✓ | |

**Table S4(c). Composition of complex nutrients in the "Sugar derivatives" class**

| Family | Name | *Ec* | *Bs* | *Sa* |
|---|---|---|---|---|
| Acetate, D-GluA* | NAc-D-GluA* | ✓ | ○ | ○ |
| Pyruvate, Acetate, D-Mannose | N-Acetylneuraminic acid | ✓ | ○ | ○ |
| Uracil, D-Ribose, D-Galactose | UDP-galactose | ○ | | |
| Acetate, D-Galactose | NAc-D-GalA** 1-phosphate | x | | |
| D-Glucose, Acetate×2, D-Lactate, D-GluA* | NAc-D-GluA* w/ NAc-muramate | ✓ | | |
| Acetate, Uracil, D-Ribose, D-GluA* | UDP-N-acetyl-D-GluA*mine | ○ | | |
| L-Lysine, D-Fructose | Psicoselysine | ○ | | |
| D-Glucose, Uracil, D-Ribose | UDP-D-glucose | ○ | | |
| D-Glucose, Saligenin | Salicin | | ○ | x |
| Uracil, D-Ribose, D-Glucuronate | UDP-glucuronate | ○ | | |
| D-Glucose, 4-Hydroxyphenol | Arbutin | | ○ | |
| Acetate, D-GluA* | NAc-D-GluA* 1-phosphate | ✓ | | |
| Acetate, D-Galactose | 2-Acetamido-2-deoxy-D-galactose | x | | |
| Acetate, Uracil, D-Ribose, D-Galactose | UDP-N-acetyl-D-GalA** | ○ | | |
| D-Glucose, Acetate, D-Lactate | N-Acetylmuramate | ✓ | | |
| Acetate, D-Mannose | 2-Acetamido-2-deoxy-D-mannose | ✓ | ○ | |
| D-Mannose, (R)-Glycerate | $\alpha$-Mannosylglycerate | ✓ | | |
| L-Lysine, D-Fructose | Fructoselysine | ○ | | |

NAc: N-Acetyl, $*$: D-Glucosamine, $**$: D-Galactosamine

**Table S4(d). Composition of complex nutrients in the "Purines" class**

| Family | Name | *Ec* | *Bs* | *Sc* | *Hp* | *Sa* | *Mt* |
|---|---|---|---|---|---|---|---|
| D-Ribose, Adenine | Adenosine | ✓ | ✓ | ○ | ○ | x | |
| D-Ribose, Adenine | Deoxyadenosine | ✓ | ✓ | x | x | | |
| D-Ribose, Adenine | ATP | | | | | | x |
| D-Ribose, Adenine | dAMP | ✓ | | | | | |
| D-Ribose, Adenine | PAP | | | ○ | | | |
| D-Ribose, Adenine | 3'-AMP | ✓ | ✓ | | | | |
| D-Ribose, Adenine | 5'-AMP | ✓ | ✓ | | | | |
| D-Ribose, Adenine | 2',3'-Cyclic AMP | ✓ | | | | | |
| D-Ribose, Guanine | Guanosine | ✓ | ✓ | ○ | ○ | | |
| D-Ribose, Guanine | Deoxyguanosine | ✓ | | x | | | |
| D-Ribose, Guanine | GTP | x | | | | | |
| D-Ribose, Guanine | GDP | x | | | | | |
| D-Ribose, Guanine | GMP | ✓ | ✓ | | | | |
| D-Ribose, Guanine | dGMP | ✓ | | | | | |
| D-Ribose, Guanine | 3'-GMP | ✓ | ✓ | | | | |
| D-Ribose, Guanine | 2',3'-Cyclic GMP | ✓ | | | | | |
| D-Ribose, Hypoxanthine | Inosine | ✓ | ✓ | ○ | | | |
| D-Ribose, Hypoxanthine | Deoxyinosine | ✓ | | x | | | |
| D-Ribose, Hypoxanthine | IMP | ✓ | | | | | |
| D-Ribose, Hypoxanthine | dIMP | ✓ | | | | | |
| D-Ribose, Xanthine | Xanthosine | ✓ | ✓ | ○ | | | |
| D-Ribose, Xanthine | XMP | ✓ | | | | | |

**Table S4(e). Composition of complex nutrients in the "Pyrimidines" class**

| Family | Name | *Ec* | *Bs* | *Sc* | *Hp* | *Sa* | *Mt* |
|---|---|---|---|---|---|---|---|
| D-Ribose, Cytosine | Cytidine | ○ | ○ | ○ | x | x | x |
| D-Ribose, Cytosine | Deoxycytidine | ○ | ○ | x | x | | |
| D-Ribose, Cytosine | CMP | ○ | ○ | | | | |
| D-Ribose, Cytosine | dCMP | ○ | | | | | |
| D-Ribose, Cytosine | 3'-CMP | ○ | ○ | | | | |
| D-Ribose, Cytosine | 2',3'-Cyclic CMP | ○ | | | | | |
| Uracil, D-Ribose | Uridine | ○ | ○ | ○ | x | x | |
| Uracil, D-Ribose | Deoxyuridine | ○ | | x | x | | |
| Uracil, D-Ribose | UMP | ○ | ○ | | | | |
| Uracil, D-Ribose | dUMP | ○ | | | | | |
| Uracil, D-Ribose | 3'-UMP | ○ | ○ | | | | |
| Uracil, D-Ribose | 2',3'-Cyclic UMP | ○ | | | | | |
| D-Ribose, Thymine | Deoxythymidine | ○ | ○ | x | x | x | |
| D-Ribose, Thymine | TMP | ○ | ○ | | | | |
| D-Ribose, Thymine | dTTP | | | x | | | |

**Table S4(f). Composition of complex nutrients in the "Amino acid derivatives" class**

| Family | Name | *Ec* | *Bs* | *Sc* | *Mt* |
|---|---|---|---|---|---|
| Glycine×2 | Peptide | | | x | |
| Glycine, L-Proline | Pro-Gly | | x | | |
| Glycine, L-Aspartate | Gly-Asp | | x | | |
| Glycine, L-Glutamine | Gly-Glu | | x | | |
| Glycine, L-Asparagine | Gly-Asn | | x | | |
| Glycine, L-Methionine | Gly-Met | | x | | |
| L-Glutamate, Glycine | Gly-Glu | | x | | |
| Glycine, L-Cysteine | Cys-Gly | ✓ | x | | |
| Glycine, L-Proline | Pro-Gly | ✓ | | | |
| Glycine, L-Alanine | Ala-Gly | | x | | |
| L-Alanine, L-Threonine | Ala-Thr | | x | | |
| L-Alanine, L-Leucine | Ala-Leu | | x | | |
| L-Glutamate, L-Alanine | Ala-Glu | | x | | |
| L-Alanine, L-Glutamine | Ala-Gln | | x | | |
| L-Alanine, L-Histidine | Ala-His | | x | | |
| L-Alanine, L-Methionine | Met-Ala | | x | | |
| L-Alanine, L-Aspartate | Ala-Asp | | x | | |
| D-Alanine×2 | D-Ala-D-Ala | ✓ | ✓ | | |
| L-Glutamate, Glycine, L-Cysteine | Glutathione | ✓ | ○ | x | x |
| L-Glutamate×2, Glycine×2, L-Cysteine×2 | Glutathione disulfide | x | ○ | x | |
| L-Alanine, L-Cysteine | Lanthionine | | x | | |
| L-Glutamate, L-Alanine, mdp† | LalaDglu-mdp† | ○ | | | |
| L-Glutamate, L-Alanine, D-Alanine, mdp† | LalaDglu-mdp†-Dala | ○ | | | |
| L-Cysteine, 2-Oxobutanoate | Cystathionine | | x | | |
| Indole, Ethanol | Indole-3-ethanol | | | x | |
| Ethanal, Indole | Indoleacetaldehyde | | | x | x |
| Acetate, L-Serine | O-Acetyl-L-serine | x | | | |
| Formate×2, L-Tyrosine×2 | N,N-Bisformyl-dityrosine | | | x | |

†: meso-2,6-diaminopimelate.

**Supplemental Table S5: Nutrient classification.**

The reconstruction for *B. subtilis* included two proteins that the organism excretes, and an antibiotic (puromycin); these were ignored.

**Table S5(a). Nutrients classified as "Cofactors."**

| | |
|---|---|
| NADP+ | Coenzyme A |
| S-Adenosyl-methionine | S-Adenosyl-homocysteine |
| Haem | Flavin mononucleotide |
| Thiamin diphosphate | Bilineurine |
| Coenzyme R | Vitamin PP |
| Cobamide (w/ Fe(III)) | Niacin |
| Riboflavin | Thiamin |
| $\beta$-Nicotinamide ribonucleotide | Folate |
| 4-Hydroxyphenol | Cob(II)alamin |
| Phosphorylcholine | Lipoate |
| Cob(I)alamin | Pantothenate |
| Choline sulfate | Thiamine monophosphate |
| myo-Inositol hexakisphosphate | Pimelate |
| S-Adenosyl-4-methylthio-2-oxobutanoate | Aerobactin |
| Cobinamide | Vitamin B12 |
| Enterobactin (no Fe(III)) | Ferrichrome (w/ Fe(III)) |
| Fe(III) Dicitrate | Enterobactin (w/ Fe(III)) |
| Ferroxamine (w/ Fe(III)) | Fe(III)hydroxamate |
| Aerobactin (no Fe(III)) | Coprogen (w/ Fe(III)) |
| Coprogen (no Fe(III)) | 2,3-Dihydroxybenzoylserine (w/ Fe(III)) |
| Fe(III) hydroxamate (no Fe(III)) | Ferrichrome (no Fe(III)) |
| Ferroxamine (no Fe(III)) | Cob(I)alamin-HBI |
| Cob(I)alamin deg. | Riboflavin deg. |
| Acetyl-cystine bimane | Bimane |
| Citrate-Mg | |

**Table S5(b). Nutrients classified as "Purines."**

| | | |
|---|---|---|
| ATP | 5'-AMP | GDP |
| GTP | PAP | IMP |
| GMP | Adenine | Adenosine |
| Guanine | Hypoxanthine | Inosine |
| Deoxyguanosine | dAMP | dGMP |
| Urate | Xanthine | Guanosine |
| Deoxyadenosine | XMP | 2'-AMP |
| 3'-AMP | Xanthosine | 2',3'-Cyclic AMP |
| Deoxyinosine | 3'-GMP | 2',3'-Cyclic GMP |
| dIMP | | |

**Table S5(c). Nutrients classified as "Pyrimidines."**

| | | |
|---|---|---|
| CMP | UMP | Uracil |
| Thymine | Deoxythymidine | dCMP |
| Orotate | Uridine | TMP |
| dUMP | Cytosine | dTTP |
| Cytidine | Deoxyuridine | Deoxycytidine |
| 3'-UMP | 2',3'-Cyclic CMP | 2',3'-Cyclic UMP |
| 2'-UMP | 2'-CMP | 3'-CMP |

**Table S5(d). Nutrients classified as "Fatty acids."**

| | | |
|---|---|---|
| Hexadecanoate | Decanoate | Dodecanoate |
| Octanoate | Tetradecanoate | Octadecanoate |
| Hexadecenoate | Hexacosanoate | Octadecenoate |
| Octadecynoate | Tetradecenoate | Butanoate |
| Hexanoate | | |

**Table S5(e). Nutrients classified as "Fatty acids related."**

| | | |
|---|---|---|
| Glycerol 3-phosphate | Glycerol | Glycerophosphocholine |
| 1(Glycerol-3-Phospho)-myo-inositol | Glycerophosphoethanolamine | Glycerol 2-phosphate |
| Glycerophosphoglycerol | Glucosyl-glycerol | Glycerophosphoserine |

**Table S5(f). Nutrients classified as "Cell boundary."**

| | |
|---|---|
| Bile acid | Ergosterol |
| Lanosterol | Fecosterol |
| Zymosterol | KDO(2)-lipid IV(A) |
| KDO2-lipid (A) | Episterol |
| Phosphatidylcholine | Phosphatidyl-myo-inositol |
| 4-Amino-4-dLarab modified core oligosaccharide lipid A | Core oligosaccharide lipid A |
| (EC antigen)x4 core oligosaccharide lipid A | Phosphoethanolamine KDO(2)-lipid A |
| Hepta-acylated core oligosaccharide lipid A | Hepta-acylated KDO(s)-lipid(A) |
| Cold-adapted KDO(2)-lipid A | (O16 antigen)x4 core oligosaccharide lipid A |
| Phthiocerol dimycoserate A | Phenol palmitic acid |
| Phenol phthiocerol dimycoserate A | Episterol ester |
| Ergosterol ester | Fecosterol ester |
| Lanosterol ester | Zymosterol ester |

**Table S5(g). Nutrients classified as "Sugars."**

| | | |
|---|---|---|
| D-Glucose | Sucrose | D-Fructose |
| D-Ribose | D-Galactose | D-Mannose |
| Xylose | Glycogen | Cellobiose |
| Maltose | D-Arabinose | Lactose |
| L-Sorbose | L-Arabinose | L-Xylulose |
| Melitose | L-Rhamnose | $\beta$-D-Galactose |
| L-Fucose | Trehalose | D-Allose |
| L-Lyxose | Chitobiose | Palatinose |
| 2'-Deoxyribose | Maltotriose | Maltohexaose |
| Maltotetraose | Melibiose | D-Psicose |
| Gentiobiose | Dextrin | Pectin |
| Starch | Amylose | 1,3-$\beta$-D-Glucan |
| Arabinan | 5'-Deoxyribose | Maltoheptaose |
| Maltopentaose | | |

**Table S5(h). Nutrients classified as "Sugars related."**

| | |
|---|---|
| UDP-D-glucose | UDP-N-acetyl-D-glucosamine |
| UDP-galactose | L-Ascorbate |
| D-Fructose 6-phosphate | D-Glucose 6-phosphate |
| D-Glucose 1-phosphate | $\alpha$-D-Ribose 5-phosphate |
| myo-Inositol | N-Acetyl-D-glucosamine |
| UDP-glucuronate | D-Glucuronate |
| UDP-N-acetyl-D-galactosamine | 2-Dehydro-3-deoxy-D-gluconate |
| D-Gluconate | N-Acetylneuraminic acid |
| D-Mannose 6-phosphate | D-Glucosamine |
| D-Galacturonic acid | 6-Phospho-D-gluconate |
| D-Glucosamine 6-phosphate | Xylitol |
| D-Mannitol | $\alpha$-D-Galactopyranose 1-phosphate |
| L-Arabitol | D-Mannose 1-phosphate |
| 2-Acetamido-2-deoxy-D-mannose | L-Idonate |
| L-Gulitol | D-Glucaric acid |
| D-Galactarate | D-Galactonic acid |
| D-Fructuronate | L-Gulono-1,4-lactone |
| 5-Dehydro-D-gluconate | 2-Acetamido-2-deoxy-D-galactose |
| Salicin | D-Galactitol |
| D-Gulitol | N-Acetylmuramate |
| Methylthio-D-ribose | D-Mannosamine |
| Methyl $\beta$-D-galactopyranoside | N-Acetyl-D-glucosamine 1-phosphate |
| D-Glucuronate 1-phosphate | Arbutin |
| 2-Deoxy-D-glucose 6-phosphate | Amygdalin |
| $\alpha$-Mannosylglycerate | L-Galactonate |
| N-Acetyl-D-galactosamine 1-phosphate | N-Acetyl-D-glucosamine w/ N-Acetylmuramate |
| Fructoselysine | Psicoselysine |
| $\alpha$-Methyl-D-glucoside | $\beta$-Methylglucoside |

**Table S5(i). Nutrients classified as "Inorganic."**

| | | |
|---|---|---|
| $H_2O$ | $O_2$ | $HPO_4{}^{-2}$ |
| $CO_2$ | $H_2O_7P_2{}^{-2}$ | $NH_4{}^+$ |
| $H_2O_2$ | $Mn^{+3}$ | $Zn^{+2}$ |
| $SO_4{}^{-2}$ | $Cu^{+2}$ | $Ca^{+2}$ |
| $H^+$ | S | $NO_2{}^-$ |
| $SO_3{}^{-2}$ | $Cl^-$ | $Co^{+2}$ |
| CHN | CO | $K^+$ |
| $NO_3{}^-$ | $H_2S$ | $CHO_3{}^-$ |
| $Ni^{+2}$ | $Mg^{+2}$ | $O_3S_2{}^{-2}$ |
| NO | $HO_{10}P_3$-4 | $N_2$ |
| $Hg_2{}^+$ | $O_2.^-$ | $HN_2O$ |
| $Na^+$ | $Cd_2{}^+$ | $CNO^-$ |
| $HO_4As^{-2}$ | CNS | $O_9P_3{}^{-3}$ |
| Pb | $H_3O_3As$ | $O_3P^{-3}$ |
| Ag | $O_2S$ | $Fe^{+2}$ |
| $Fe^{+3}$ | $MoO_4{}^{-2}$ | $H_2O_4Cr$ |
| Sb | $H_2$ | $O_4W^{-2}$ |

**Table S5(j). Nutrients classified as "Amino acids."**

| | | |
|---|---|---|
| L-Glutamate | Glycine | L-Alanine |
| L-Lysine | L-Aspartate | L-Arginine |
| L-Glutamine | L-Serine | L-Methionine |
| L-Tryptophan | L-Phenylalanine | L-Tyrosine |
| L-Cysteine | L-Leucine | D-Alanine |
| L-Histidine | L-Proline | L-Asparagine |
| L-Valine | L-Threonine | D-Glutamate |
| L-Isoleucine | D-Serine | D-Cysteine |
| D-Methionine | | |

**Table S5(k). Nutrients classified as "Amino acids related."**

| | |
|---|---|
| Glutathione | L-Ornithine |
| Glutathione disulfide | 1,4-Butanediamine |
| Agmatine | Chorismic acid |
| L-homoserine | Spermidine |
| L-Carnitine | L-Citrulline |
| Indole | Tyramine |
| L-Cystine | Shikimate |
| L-Cysteate | Cystathionine |
| PACT | Trimethylamine |
| Phenylacetaldehyde | Indoleacetaldehyde |
| 4-Hydroxyphenylacetate | (3S)-3-Methyl-2-oxopentanoate |
| meso-2,6-Diaminoheptanedioate | Glycine betaine |
| Spermine | Indoleacetate |
| Indole-3-ethanol | O-Acetyl-L-serine |
| D-Ala-D-Ala | 3-Phosphoserine |
| Trimethylamine N-oxide | Cys-Gly |
| Cadaverine | L-Pyroglutamic acid |
| D-O-Phosphoserine | L-Methionine S-Oxide |
| S-Methyl-L-Methionine | 3,4-Dihydroxyphenethylamine |
| 4-Hydroxyphenylacetaldehyde | 3,4-Dihydroxyphenylacetaldehyde |
| Phenylethylamine | Phenylpropanoate |
| Phenylethyl alcohol | Phosphoarginine |
| Ectoine | Phosphonotyrosine |
| L-Djenkolic acid | Proline betaine |
| Dihydro-3-coumaric acid | L-Threonine O-3-phosphate |
| 3-Hydroxycinnamic acid | MOPS |
| L-Methionine R-Oxide | Lanthionine |
| Gly-Glu | Gly-Asn |
| Ala-Thr | Ala-Leu |
| Ala-His | Ala-Gly |
| Ala-Glu | Ala-Gln |
| Pro-Gly | Gly-Asp |
| Met-Ala | Gly-Met |
| Gly-Glu | Ala-Asp |
| Arseno-betaine | Peptide |
| LalaDglu-meso-2,6-diaminoheptanedioate | LalaDglu-meso-2,6-diaminoheptanedioate-Dala |
| $\gamma$-Butyro-betaine | 4-hydroxy-5-methyl-3-2H-furanone |
| L-Methionine-R-Sulfoxide | L-Methionine-S-Sulfoxide |
| Pro-Gly | N,N-Bisformyl-dityrosine |

**Table S5(l). Nutrients classified as “Organic compounds.”**

| | | |
|---|---|---|
| Pyruvate | $\alpha$-Ketoglutarate | Acetate |
| Oxaloacetate | Succinate | Glyoxylate |
| Formate | Formalin | Phosphoenolpyruvate |
| Ethanal | Urea | 3-Aminopropionate |
| Fumarate | Methyl alcohol | L-Malate |
| Citrate | Glycolate | Propionate |
| Acetoacetate | Carbamoyl-phosphate | Glycerone |
| (S)-Lactate | Aminoethanol | 3-Phospho-DL-glycerate |
| Taurine | D-Lactate | (R)-Glycerate |
| Glycolaldehyde | Isocitrate | 4-Aminobutyrate |
| O-Phosphorylethanolamine | Methanethiol | 5-Aminolevulinate |
| (R)-Dimethylketol | Ethanol | Propionaldehyde |
| (R)-Malate | Allantoic acid | (CH3)2NH |
| meso-Tartarate | 4-Aminobenzoate | D-Glyceraldehyde |
| Methyl sulfide | (S)-Propane-1,2-diol | 2-Phospho-D-glycerate |
| 2,3-Butanedione | D-Tartrate | (S)-2-Hydroxy-2-methyl-3-oxobutanoate |
| Phosphoglycolate | 7-8-Diaminononanoate | 8-Amino-7-oxononanoate |
| 4-Trimethylammoniobutanoate | Methane | Oxamate |
| Allantoin | 2-Propenamide | Mercaptoacetate |
| 2-Methylbutanal | Saligenin | 3-Carboxy-3-hydroxyisocaproate |
| (R)-Propane-1,2-diol | (R,R)-2,3-Butanediol | 2-Hydroxyethanesulfonate |
| 2-Hydroxybutyrate | Acetamide | 2,3-Diaminopropionate |
| Aminoacetaldehyde | Isopentyl alcohol | Isovaleraldehyde |
| Dimethyl sulfoxide | Methanesulfonate | Crotono-betaine |
| Butyro-betaine | Isoamyl acetate | Phenethyl acetate |
| Sulfoacetic acid | Isobutyl alcohol | Hexanesulfonate |
| Ethanesulfonate | Butanesulfonate | 3-Aminobutanoate |
| Acetyl ester | 4-hydroxybenzyl alcohol | Monomethylamine |
| 2-Methylbutyl acetate | 2-Methyl-1-butanol | 2-Methylpropanal |
| Isobutyl acetate | | |

**Supplemental Table S6: Uptake of Sugars, Sugar derivatives, Fatty acids, and Bases.**

The presence of a symbol indicates that a species uptakes the nutrient in question. The symbol type indicates whether the uptaken nutrient can be a source of carbon in the species in question: (✓) source of carbon, (x) not a source of carbon.

**Table S6(a). Purines.**

| Name | *Bs* | *Ec* | *Mb* | *Sc* |
|---|---|---|---|---|
| Adenine | ✓ | ✓ | | x |
| Guanine | ✓ | ✓ | | x |
| Xanthine | ✓ | ✓ | | x |
| Hypoxanthine | ✓ | ✓ | | x |
| Urate | ✓ | | | |

**Table S6(b). Pyrimidines.**

| Name | *B. subtilis* | *E. coli* | *M barkeri* | *S. cerevisiae* |
|---|---|---|---|---|
| Uracil | x | x | | x |
| Cytosine | x | x | | x |
| Thymine | x | x | | x |
| Orotate | | x | | |

**Table S6(c). Fatty acids.**

| Name | *B. subtilis* | *E. coli* | *M barkeri* | *S. cerevisiae* |
|---|---|---|---|---|
| Butanoate | | ✓ | | |
| Hexanoate | | ✓ | | |
| Octanoate | | ✓ | | |
| Decanoate | | ✓ | | x |
| Dodecanoate | | ✓ | | x |
| Tetradecanoate | | ✓ | | x |
| Tetradecenoate | | ✓ | | |
| Hexadecanoate | | ✓ | | x |
| Hexadecenoate | | ✓ | | x |
| Octadecanoate | | ✓ | | x |
| Octadecenoate | | ✓ | | x |
| Octadecynoate | | | | x |
| Hexacosanoate | | | | x |

**Table S6(d). Sugars.**

| Name | *B. subtilis* | *E. coli* | *M barkeri* | *S. cerevisiae* |
|---|---|---|---|---|
| D-Glucose | ✓ | ✓ | | ✓ |
| D-Mannose | ✓ | ✓ | | ✓ |
| D-Fructose | ✓ | ✓ | | ✓ |
| D-Arabinose | ✓ | | | x |
| D-Galactose | ✓ | ✓ | | ✓ |
| D-Allose | | ✓ | | |
| $\beta$-D-Galactose | | ✓ | | |
| D-Ribose | ✓ | ✓ | | ✓ |
| 2'-Deoxyribose | ✓ | | | |
| 5'-Deoxyribose | | | | |
| L-Fucose | | ✓ | | |
| L-Xylulose | | ✓ | | |
| L-Lyxose | | ✓ | | |
| L-Rhamnose | ✓ | ✓ | | |
| L-Sorbose | ✓ | | | x |
| L-Arabinose | ✓ | ✓ | | x |
| Sucrose | ✓ | ✓ | | ✓ |
| Lactose | ✓ | ✓ | | |
| Maltose | ✓ | ✓ | | ✓ |
| Melibiose | ✓ | ✓ | | ✓ |
| Trehalose | ✓ | ✓ | | ✓ |
| Cellobiose | ✓ | | | |
| Palatinose | ✓ | | | |
| Chitobiose | x | | | |
| Glycogen | ✓ | | | |
| Melitose | ✓ | | | |
| Starch | ✓ | | | |
| Dextrin | ✓ | | | |
| Arabinan | ✓ | | | |
| Xylose | ✓ | ✓ | | ✓ |
| Maltotriose | ✓ | ✓ | | |
| Maltotetraose | | ✓ | | |
| Maltopentaose | | ✓ | | |
| Maltohexaose | | ✓ | | |
| Amylose | | ✓ | | |
| 1,3-$\beta$-D-Glucan | | | | ✓ |
| Pectin | | | | x |

**Table S6(e). Sugar derivatives.**

| Name | *B. subtilis* | *E. coli* | *M barkeri* | *S. cerevisiae* |
|---|---|---|---|---|
| Xylitol | | | | ✓ |
| L-Arabitol | ✓ | | | x |
| L-Gulitol | ✓ | ✓ | | ✓ |
| D-Gulitol | | | | x |
| D-Mannitol | ✓ | ✓ | | |
| D-Galactitol | ✓ | ✓ | | |
| $\alpha$-D-Ribose 5-phosphate | | ✓ | | |
| D-Fructose 6-phosphate | | ✓ | | |
| D-Glucose 6-phosphate | ✓ | ✓ | | |
| D-Glucose 1-phosphate | ✓ | ✓ | | |
| D-Glucuronate 1-phosphate | | ✓ | | |
| D-Mannose 1-phosphate | ✓ | | | |
| D-Mannose 6-phosphate | ✓ | ✓ | | |
| D-Glucosamine 6-phosphate | ✓ | ✓ | | ✓ |
| $\alpha$-D-Galactopyranose 1-phosphate | | ✓ | | |
| 6-Phospho-D-gluconate | ✓ | | | |
| 5-Dehydro-D-gluconate | | ✓ | | |
| 2-Dehydro-3-deoxy-D-gluconate | ✓ | ✓ | | |
| D-Gluconate | ✓ | ✓ | x | |
| D-Glucuronate | ✓ | ✓ | | |
| D-Glucaric acid | ✓ | ✓ | | |
| D-Glucosamine | ✓ | ✓ | | |
| D-Fructuronate | | ✓ | | |
| D-Galactarate | ✓ | ✓ | | |
| D-Galacturonic acid | ✓ | ✓ | | x |
| D-Galactonic acid | | ✓ | | |
| L-Galactonate | | ✓ | | |
| L-Idonate | | ✓ | | |
| L-Ascorbate | | ✓ | | |
| myo-Inositol | ✓ | x | | x |
| Methylthio-D-ribose | x | | | |
| $\alpha$-Methyl-D-glucoside | ✓ | | | |
| $\beta$-Methylglucoside | ✓ | | | |

**Supplemental Table S7: $G$ and $NG$ nutrients in pathways considered for the logistic model.**
The data in this table complements Figure S2.

**Table S7. $G$ and $NG$ nutrients in candidate pathways.**

| Model | Pathway | $G$ | $NG$ | $G^*$ | $NG^*$ |
|---|---|---|---|---|---|
| 1 | Glyoxylate and dicarboxylate metabolism | 13 | 2 | 13 | 2 |
| 2 | Glycerolipid metabolism | 10 | 1 | 8 | 1 |
| 3 | Alanine, aspartate and glutamate metabolism | 11 | 1 | 7 | 1 |
| 4 | Glycolysis / Gluconeogenesis | 9 | 2 | 6 | 2 |
| 5 | Arginine and proline metabolism | 14 | 5 | 7 | 4 |
| 6 | Glycine, serine and threonine metabolism | 12 | 5 | 7 | 5 |
| 7 | Propanoate metabolism | 6 | 2 | 4 | 2 |
| 8 | Methane metabolism | 6 | 5 | 3 | 4 |
| 9 | Phenylalanine metabolism | 7 | 9 | 3 | 8 |
| 10 | Purine metabolism | 5 | 1 | 2 | 0 |
| 11 | Vitamin B6 metabolism | 4 | 1 | 2 | 0 |
| 12 | Butanoate metabolism | 9 | 5 | 2 | 4 |
| 13 | Pyruvate metabolism | 9 | 4 | 1 | 1 |
| 14 | Porphyrin and chlorophyll metabolism | 4 | 1 | 1 | 0 |
| 15 | Cysteine and methionine metabolism | 7 | 12 | 1 | 10 |
| 16 | Valine, leucine and isoleucine biosynthesis | 2 | 11 | 0 | 8 |
| 17 | Lysine degradation | 1 | 5 | 0 | 5 |
| 18 | Tyrosine metabolism | 4 | 7 | 0 | 4 |
| 19 | Taurine and hypotaurine metabolism | 4 | 4 | 0 | 3 |
| 20 | Phenylalanine, tyrosine and tryptophan biosynthesis | 1 | 7 | 0 | 3 |
| 21 | Biotin metabolism | 0 | 3 | 0 | 2 |
| 22 | Folate biosynthesis | 0 | 4 | 0 | 1 |
| 23 | beta-Alanine metabolism | 2 | 5 | 0 | 1 |
| 24 | Tryptophan metabolism | 0 | 3 | 0 | 1 |
| 25 | Glycerophospholipid metabolism | 3 | 2 | 0 | 1 |

$G^*$ and $NG^*$ are the numbers of $G$ and $NG$ nutrients respectively that are not included in the previous pathways. For example, Cysteine and methionine metabolism only has one $G$ nutrient out of seven that are not included in the previous 14 pathways.

**Table S8. Coefficients for the logistic model.**

| **Pathway** | $\beta$ |
|---|---|
| Constant ($\beta_0$) | $-1.46$ |
| Alanine, aspartate and glutamate metabolism | 3.84 |
| Arginine and proline metabolism | 1.78 |
| Folate biosynthesis | $-16.43$ |
| Glycerolipid metabolism | 2.85 |
| Glycine, serine and threonine metabolism | 1.92 |
| Glycolysis / Gluconeogenesis | 1.78 |
| Glyoxylate and dicarboxylate metabolism | 2.49 |
| Propanoate metabolism | 3.18 |
| **Interaction** | $\gamma$ |
| Arginine and proline metabolism:$\beta$-Alanine metabolism | $-3.15$ |
| Propanoate metabolism:Pantothenate and CoA biosynthesis | $-3.33$ |

# References


[1] **Wong, W. W., L. M. Tran, and J. C. Liao**, 2009. A hidden square-root boundary between growth rate and biomass yield. Biotechnol Bioeng 102:73–80.

[2] **Feist, A. M., C. S. Henry, J. L. Reed, M. Krummenacker, A. R. Joyce, P. D. Karp, L. J. Broadbelt, V. Hatzimanikatis, and B. Ø. Palsson**, 2007. A genome-scale metabolic reconstruction for *Escherichia coli* K-12 MG1655 that accounts for 1260 ORFs and thermodynamic information. Mol. Syst. Biol. 3:121.

[3] **Neidhardt, F., J. Ingraham, and M. Schaechter**, 1990. Physiology of the bacterial cell: a molecular approach. Sinauer Associates, Sunderland, MA.

[4] **Förster, J., I. Famili, P. Fu, B. Ø. Palsson, and J. Nielsen**, 2003. Genome-scale reconstruction of the saccharomyces cerevisiae metabolic network. Genome Res 13:244–253.

[5] **Verduyn, C., A. H. Stouthamer, W. A. Scheffers, and J. P. van Dijken**, 1991. A theoretical evaluation of growth yields of yeasts. Antonie Van Leeuwenhoek 59:49–63.

[6] **Thiele, I., T. D. Vo, N. D. Price, and B. Ø. Palsson**, 2005. Expanded metabolic reconstruction of *Helicobacter pylori* (iIT341 GSM/GPR): an in silico genome-scale characterization of single- and double-deletion mutants. J. Bacteriol. 187:5818–5830.
